# Advancing targeted protein degradation: pLIRTAC’s role in glioma and CAR-T cell therapy

**DOI:** 10.1080/15548627.2026.2668653

**Published:** 2026-05-07

**Authors:** Kunjian Lei, Yishuang Li, Zhihong Zhou, Jingying Li, Linzhen Huang, Wenji Liu, Kai Huang, Xingen Zhu

**Affiliations:** a Department of Neurosurgery, The Second Affiliated Hospital, Jiangxi Medical College, Nanchang University, Nanchang, Jiangxi, P. R. China; b School of Basic Medical Sciences, Nanchang University, Nanchang, P. R. China; c Jiangxi Province Key Laboratory of Neurological Diseases, Nanchang, Jiangxi, P. R. China; d JXHC Key Laboratory of Neurological Medicine, Nanchang University, Nanchang, Jiangxi, China; e The MOE Basic Research and Innovation Center for the Targeted Therapeutics of Solid Tumors, Jiangxi Medical College, Nanchang University, Nanchang, Jiangxi, China; f Department of Comprehensive Intensive Care Unit, The Second Affiliated Hospital of Nanchang University, Nanchang, P. R. China

**Keywords:** CAR-T, pLIRTAC, selective autophagy, T-cell exhaustion, targeted protein degradation, tumor therapy

## Abstract

The rapid development of targeted protein degradation (TPD) has shown profound effects on disease treatment. Precise and effective targeted degradation tools that target endogenous proteins are essential to accelerate advances in treatment methods. Selective macroautophagy/autophagy relies on the activity of related receptors to achieve the degradation of specific intracellular components in lysosomes, but the methodology of selective autophagy for tumor therapy and chimeric antigen receptor (CAR)-T cell modification is yet unexplored. Here, we developed a peptide-based LC3-interacting region-targeting chimera (pLIRTAC) that accurately and efficiently targeted the degradation of AKT1 for glioma treatment. pLIRTAC could also inhibit the development of tumor cells by *in vitro* delivery after purification. For CAR-T cell therapy, pLIRTAC could significantly improve the efficacy of CAR-T cell-targeted lysis of tumor cells both *in vitro* and *in vivo*. pLIRTAC binds to autophagy-associated proteins through LC3-interacting region (LIR) motifs and to target proteins through protein-targeting short peptides, and targets the protein of interest (POI) based on the selective autophagy lysosomal pathway. pLIRTAC has been remarkably successful both *in vivo* and *in vitro*, providing a robust and effective tool for the control of endogenous abnormal proteins in cells, and can potentially further expand the therapeutic application of TPD technology.

**Abbreviation:** ATG8s: mammalian Atg8 (autophagy related 8)-family proteins; Baf-A1: bafilomycin A_1_; CAR: chimeric antigen receptor; CQ: chloroquine; CRISPR: clustered regularly interspaced short palindromic repeats; EBSS: Earle’s balanced salt solution; LIR: LC3-interacting region; 3 MA: 3-methyladenine; MFI: mean fluorescence intensity; pLIRTAC: peptide-based LC3-interacting region-targeting chimera; POI: protein of interest; PROTAC: proteolysis-targeting chimera; SARS: selective autophagy receptors; TPD: targeted protein degradation.

## Introduction

The TPD strategy has been attracting increasing attention since it was proposed, with related technologies continuously evolving to the extent that it is considered a drug development method with infinite potential [1,2]. In contrast to strategies that primarily focus on directly regulating protein activity, the TPD strategy harnesses the cell’s inherent mechanisms of destruction to eliminate specific disease-associated proteins [3]. The TPD strategy primarily operates through two protein degradation pathways within cells: the proteasome and lysosome pathways [4,5]. Reportedly, numerous highly promising TPD drugs have been developed based on the proteasome pathway, such as proteolysis-targeting chimera (PROTAC), molecular glue, and many PROTAC-based technologies [6–10]. However, the limitations of the proteasome pathway are significant, including the need to identify specific E3 ligases for targeting proteins and suitable E3 ligase ligands, as well as the restriction to degrade only intracellular proteins [1]. In contrast to the proteasome pathway, the lysosome pathway can degrade unnecessary or damaged proteins inside and outside of cells, as well as damaged organelles, and is accomplished through various processes such as endocytosis, phagocytosis, and/or autophagy [11–13]. In the selective autophagy process, with the assistance of LIR, “cargo” deemed unnecessary or damaged can be recognized by selective autophagy receptors (SARs) and form autophagosomes, which are ultimately transported to the lysosome for degradation [14,15]. Consequently, selective autophagy holds great promise for removing disease-causing proteins or certain functionally inhibitory proteins.

With the advancement of nucleotide sequencing and protein identification technologies, an increasing number of disease-associated proteins have been identified, and researchers have further focused on the treatment strategies for diseases based on the regulation of these proteins [16–19]. For instance, targeted degradation of AKT has shown significant and long-lasting inhibition of various malignant tumors, including gliomas [20,21]. Inhibiting the aggregation of Tau protein can improve the progression of Alzheimer disease, while reducing the overexpression of *DAPK1* can mitigate brain damage caused by cerebral ischemia [22,23]. Additionally, inhibiting overexpressed *KDM5B* can suppress H3K4 methylation levels to restrain the malignant behavior of stem cells and cancer cells [24]. To selectively target these “abnormal” proteins within cells, we have utilized the highly selective and small-sized LIR motif in this study, and combined it with short peptides that can specifically bind to target proteins, to create a targeted chimeric molecule capable of specifically degrading target proteins. In our study, targeted degradation of KDM5B, AKT1, and BATF has shown significant effectiveness, with the degradation effect being notably superior on cytoplasmic proteins than on nuclear proteins.

CAR-T cell immunotherapy has shown significant tumor treatment efficacy with higher accuracy and greater sensitivity than other targeted therapies that specifically degrade target proteins [25]. Therefore, CAR-T cell immunotherapy has achieved several meaningful breakthroughs in a short period of time. However, researchers have also discovered T-cell exhaustion as a major obstacle in CAR-T cell and other anti-tumor immunotherapies [26,27]. The key genes influencing T-cell exhaustion are *BATF*, *IKZF2*, *TLE4*, *TGFBR2, ADORA2A, Fli1, TOX*, and *Arid1a* [28–31]. Designing degradation tools targeting these proteins is highly likely to inhibit T cell exhaustion and further enhance the efficacy of CAR-T cell anti-tumor therapy. In most cases of CAR-T cell engineering, T cells often require input from the clustered regularly interspaced short palindromic repeats (CRISPR) system during the process of introducing CAR into the genome. This may increase the difficulty of screening CAR-T cells and suppress the activity of CAR-T cells. Therefore, we designed a strategy to co-express a CAR and a peptide sequence targeting a specific exhaustion-associated protein for degradation (pLIRTAC) via a P2A peptide at the CAR C terminus. This approach successfully reduced the number of transfection cycles and screening time, and markedly enhanced the persistence of CAR-T cells.

In this study, we designed a tool called pLIRTAC based on LIR and protein-targeting peptides, which can be used to degrade intracellular proteins. This tool can rapidly and efficiently degrade intracellular target proteins and can also be used to screen for LIR motifs with superior targeting degradation capabilities. Through *in vivo* and *in vitro* applications, we determined that pLIRTAC can significantly degrade target proteins and influence their impact on cell function. In tumor treatment studies, pLIRTAC can not only be directly applied to degrade target proteins to inhibit the occurrence and development of tumors, but can also enhance the effectiveness of immunotherapy by targeting the exhaustion of CAR-T cell-related proteins. Overall, it provides a simple and effective drug development tool by greatly supplementing the application of TPD strategies in disease treatment and hence presents a potentially promising therapeutic method.

## Results

### LC3-interacting regions effectively induce fusion protein degradation

**Figure 1.**
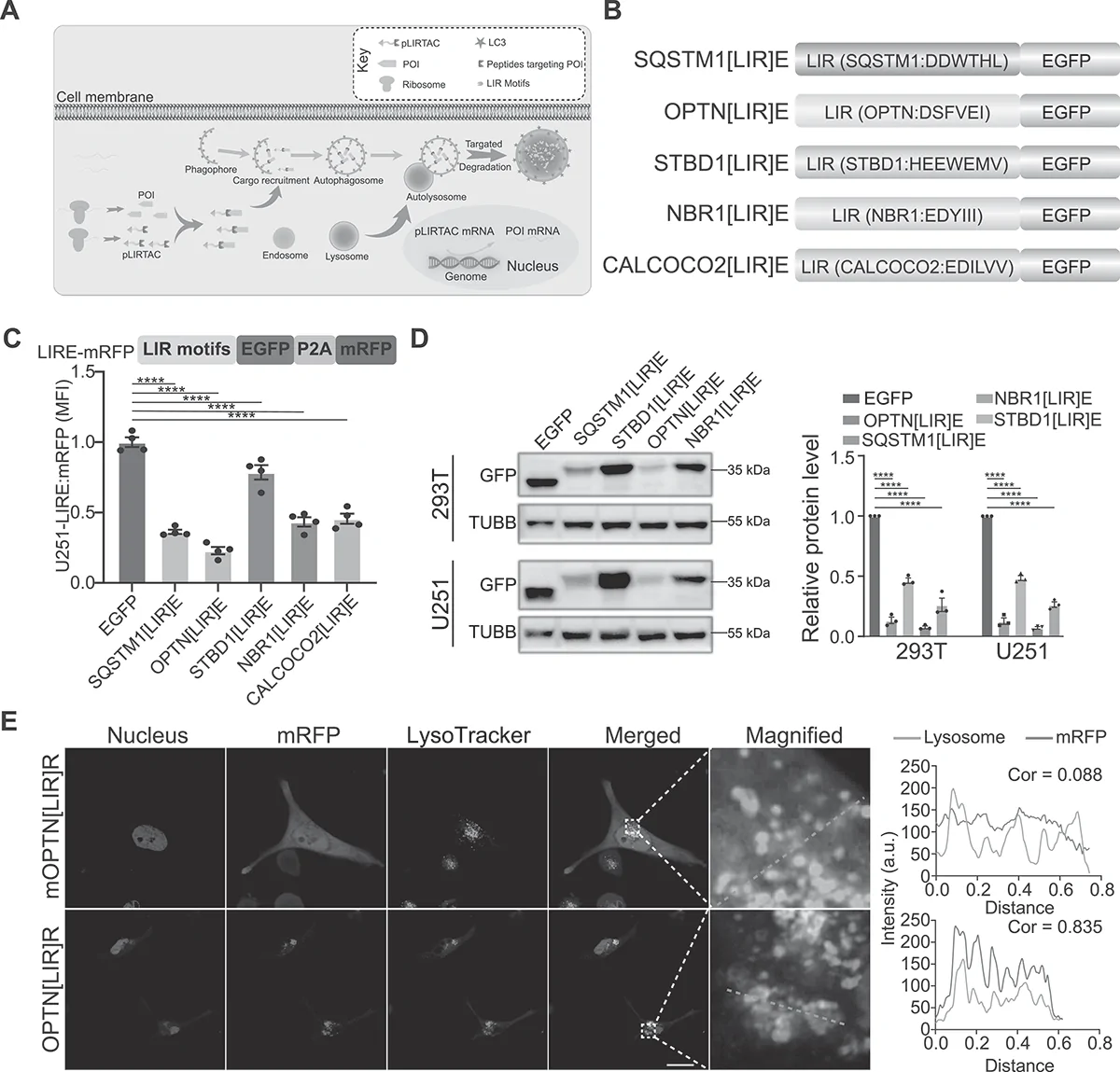

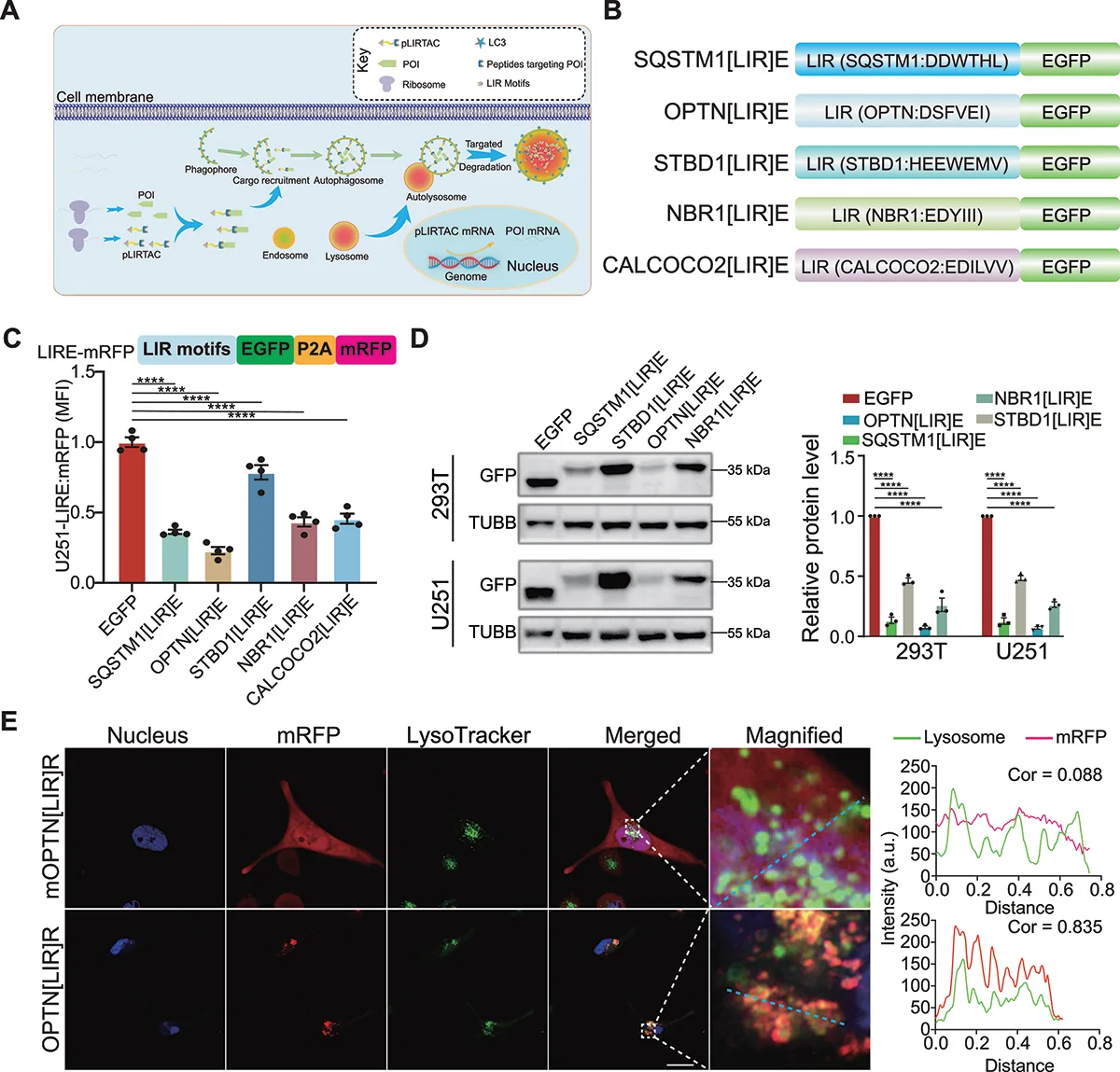
OPTN[LIR]E can be degraded with high efficiency. (A) Schematic diagram of intracellular pLIRTAC targeted degradation of poi. (B) The LIR motif from a variety of proteins was recombined with EGFP to obtain the schematic structure of the recombinant protein. (C) TOP, schematic diagram of the constructed LIRE-P2A-EGFP plasmid. Bottom, relative ratio of the mean fluorescence intensity (MFI) of different lire variants to their corresponding mRFP (1 × 10^4^ cells per group, biologically replicated three times). (D) Left, TUBB as an internal reference, the protein expression levels of four lire were detected using GFP antibodies. Right, after standardizing four types of lire, their intracellular protein levels were quantitatively displayed. (E) Expression and colocalization of mOPTN[LIR]R and OPTN[LIR]R with lysosomes in U251 cells (63 ×). Scale bar: 20 μm. Correlation (cor) ≥0.7 indicates strong correlation, 0.4 < cor ≤0.7 indicates moderate correlation, 0.2 < cor ≤0.4 indicates weak correlation, and cor ≤0.2 indicates almost no correlation.

Selective autophagy is a process in which specific cellular components are degraded in autophagosomes, late endosomes, or lysosomes through the activity of SARs [32,33]. SARs interact with mammalian Atg8 (autophagy related 8)-family proteins (ATG8s) involved in autophagy through LIR motifs, which are conserved in vertebrates [5,34,35]. The pLIRTAC developed in this study can efficiently degrade specific target proteins via selective autophagy pathways (Figure 1(A)). Currently, many SARs such as SQSTM1/p62, STBD1, NBR1, CALCOCO2/NDP52, and OPTN have been identified, each of which participates in different pathways and binds to different substrates [33,36]. In our study, we selected the LIR motifs of SQSTM1, STBD1, NBR1, CALCOCO2, and OPTN and directly linked them to the N terminus of EGFP to obtain recombinant proteins, namely SQSTM1-EGFP (SQSTM1[LIR]E), STBD1-EGFP (STBD1[LIR]E), NBR1-EGFP (NBR1[LIR]E), CALCOCO2-EGFP (CALCOCO2[LIR]E), and OPTN-EGFP (OPTN[LIR]E), with wild-type EGFP (EGFP) as a control (Figure 1(B)). We evaluated the efficiency of different LIR motifs in inducing the degradation of recombinant proteins by assessing the amount of green fluorescence and signal changes. First, we further constructed the LIRE-mRFP plasmid and successfully obtained high levels of expression of EGFP, SQSTM1[LIR]E, STBD1[LIR]E, NBR1[LIR]E, CALCOCO2[LIR]E, and OPTN[LIR]E in 293T and U251 cells based on a transient overexpression system. Next, we utilized flow cytometry to detect the intensity of green fluorescence and red fluorescence in the cells after 24 h of overexpression and calculated the mean fluorescence intensity (MFI) of each group. OPTN[LIR]E group had a significantly lower ratio of LIRE: mRFP than the other groups (Figure 1(C), S1A). We also calculated the integrated density of green fluorescent cells expressing LIRs-EGFP (Figure S1B). Given that OPTN[LIR]E demonstrated the greatest potential among all LIR-EGFP constructs, we introduced mutations into the LIR motif of OPTN[LIR]E (DSFVEI → DSAVEA) and designated the resulting construct as mut-OPTN[LIR]E (mOPTN[LIR]E). We further detected the intracellular protein levels of LIRs-EGFP and mOPTN[LIR]E, and found that OPTN[LIR]E had the lowest expression level, although the intracellular steady-state levels of the five LIR recombinant proteins were significantly lower than that of mOPTN[LIR]E. (Figure 1(D), S1C). However, there was no significant change observed in the mRNA levels of LIRs-EGFP, which further confirmed that the protein level of OPTN[LIR]E was downregulated after transcriptional translation (Figure S1D). To more clearly capture the specific distribution of LIR recombinant proteins in cells, and considering that EGFP undergoes quenching in the acidic environment of lysosomes [37], we further established plasmids capable of expressing mut-OPTN-mRFP (mOPTN[LIR]R) or OPTN-mRFP (OPTN[LIR]R) (Figure S1E). To observe the subcellular localization distribution of OPTN[LIR]R, we captured significant co-localization between OPTN[LIR]R and lysosomes marker (LAMP1) under high magnification in U87 (correlation = 0.755), U251 (correlation = 0.835), and 293T (correlation = 0.873) cells (63 ×) (Figure 1(E), S1F-G), which was markedly different from that of mOPTN[LIR]R. Next, we validated the content of intracellular OPTN[LIR]R relative to mOPTN[LIR]R and obtained results similar to those of EGFP-based recombinant proteins (Figure S1H), with OPTN[LIR]R showing significantly lower integrated density. Interestingly, while OPTN[LIR]R was significantly reduced, the EGFP used as a control did not change significantly (Figure S1I-J). These results indicated that both EGFP and mRFP can be significantly induced to degrade with the help of some LIR motifs, and the degradation pathway is probably related to lysosomes.

To investigate whether the number of LIR motif repeats affects the degradation efficiency of recombinant proteins, we designed an OPTN[LIR]E variant containing two tandem LIR motifs (2 × OPTN[LIR]E) and introduced the corresponding mutation (2 × mOPTN[LIR]E, DSFVEIDSFVEI to DSAVEADSAVEA). Notably, 2 × OPTN[LIR]E exhibited a significantly lower steady-state level compared to 1 × OPTN[LIR]E, and both variants showed markedly reduced expression relative to their corresponding mutants (Figure S2A-B).

### OPTN[LIR]E is degraded intracellularly via selective autophagy

**Figure 2.**
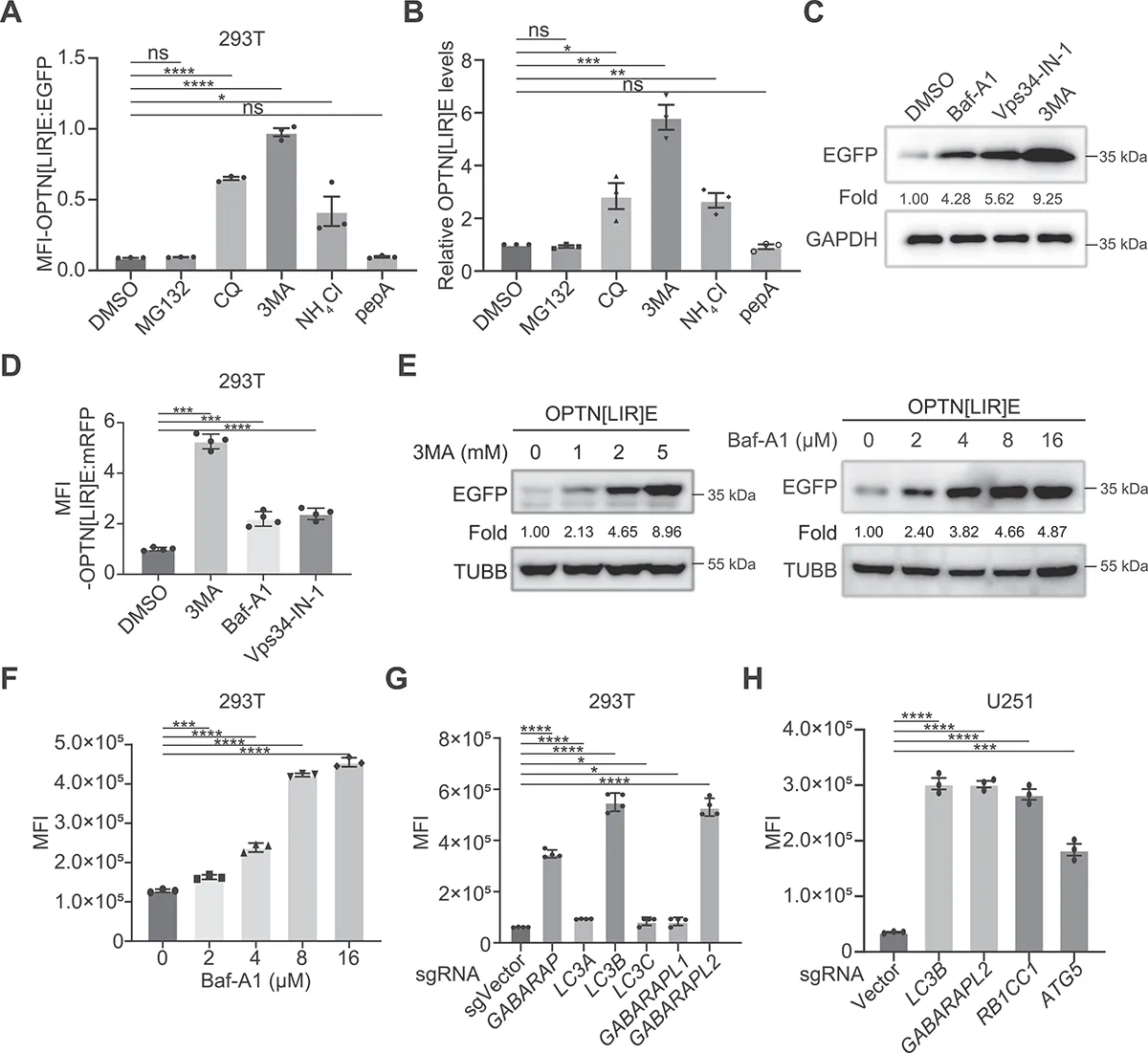

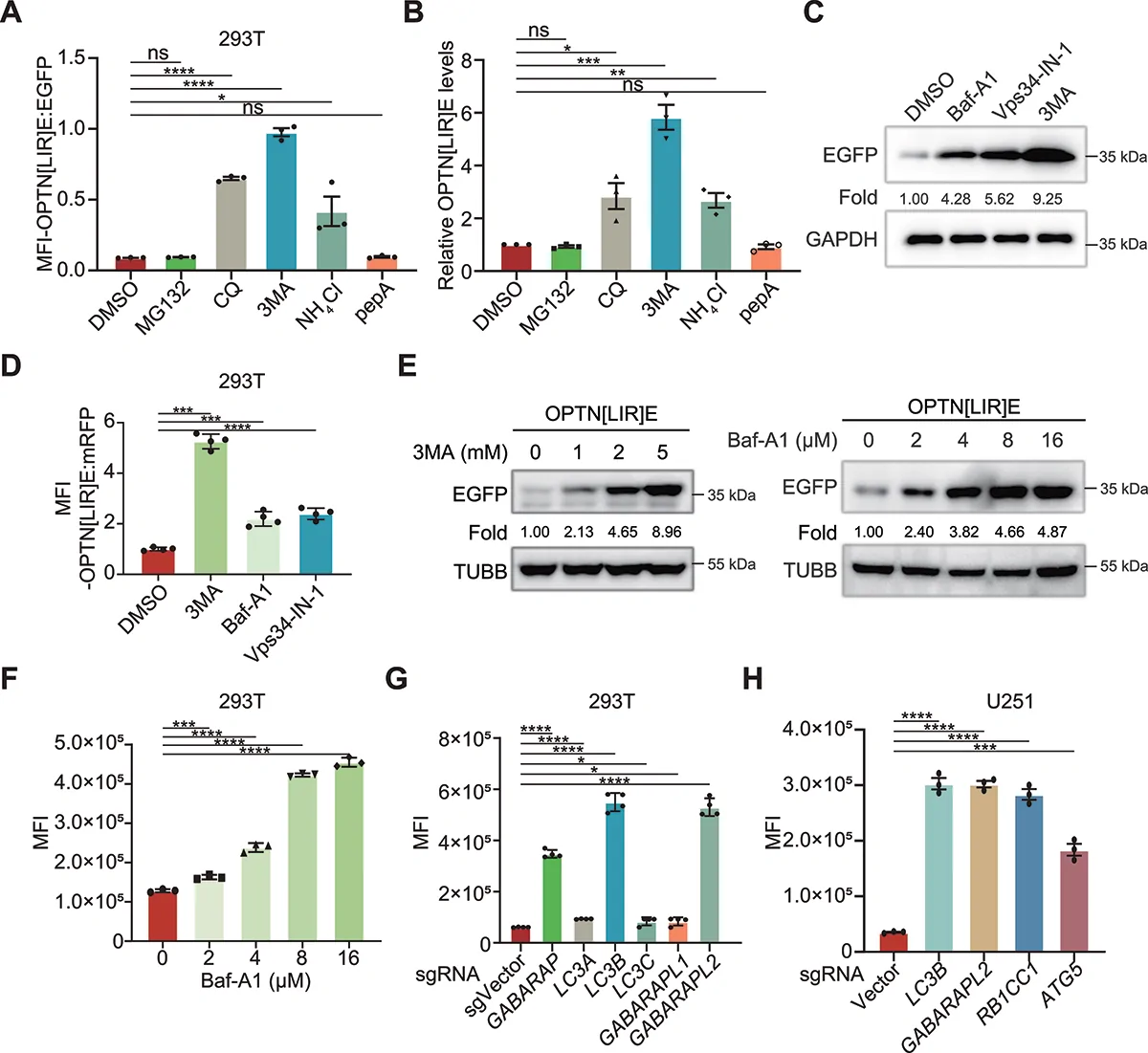
OPTN[LIR]E can be degraded through the autophagy-lysosomal pathway. (A) Differences in the MFI, the ratio of OPTN[LIR]E to EGFP in 293T cells overexpressing OPTN[LIR]E or EGFP treated with DMSO, MG132 (4 μM), CQ (500 nM), 3 MA (5 mM), NH_4_Cl (20 mM), and pepA (1 μM) for 24 h. (B) Relative protein levels of OPTNE in 293T cells overexpressing OPTN[LIR]E treated with DMSO, MG132, CQ, 3 MA, NH_4_Cl, and pepA for 24 h. Protein level (C) and MFI ratio (D) Difference in 293T cells expressing OPTN[LIR]E after 24 h of treatment with DMSO, Baf-A1 (10 μM), Vps34-IN-1 (5 μM), or 3 MA. (E) Protein level changes in 293T cells expressing OPTN[LIR]E after 24 h of treatment with different concentration gradients of 3 MA (left) or Baf-A1 (right). (F) MFI difference in 293T cells expressing OPTN[LIR]E after 24 h of treatment with different concentration gradients of Baf-A1. (G) The changes in MFI in the OPTN[LIR]E group relative to the control group (empty vector as control) after knocking out *GABARAP*, *GABARAPL1*, *GABARAPL2*, *LC3A*, *LC3B*, or *LC3C* in 293T cells. (H) The changes in MFI in the OPTN[LIR]E group relative to the control group after knocking out *RB1CC1* or *ATG5* in 293T cells.

Currently, TPD via the lysosomal pathway mainly relies on autophagy to regulate the intracellular degradation of proteins and organelles, with autophagy encompassing three main forms: autophagy, microautophagy, and chaperone-mediated autophagy/CMA [38,39]. To further elucidate the specific degradation pathway of LIR recombinant proteins, we treated EGFP and OPTN[LIR]E with various proteasome and lysosomal pathway inhibitors. The results showed that 3-methyladenine (3 MA, a PtdIns3K inhibitor commonly used to suppress the early stages of autophagy), chloroquine (CQ; a lysosomal acidification inhibitor that blocks autophagosome-lysosome fusion, thereby inhibiting late-stage autophagy), and NH_4_Cl (a weak base that neutralizes lysosomal acidity, inhibiting autophagosome degradation) can significantly inhibit the degradation of OPTN[LIR]E instead of EGFP (Figure 2(A), S2C). Next, we performed single-cell fluorescence and integrated density analysis of OPTN[LIR]E in 293T and U251 cells. The results revealed that 3 MA had a particularly pronounced inhibitory effect on OPTN[LIR]E degradation (Figure S2D). Protein-level analysis further confirmed this conclusion (Figure 2(B), S2E). Additionally, we also found that 3 MA, CQ, and NH_4_Cl could significantly reverse the degradation of OPTN[LIR]R without affecting EGFP levels (Figure S2F). In addition, given the unique inhibitory effects of bafilomycin A_1_ (Baf-A1) and VPS34-IN-1 on distinct steps of the autophagy pathway – Baf-A1 inhibits autophagosome-lysosome fusion, while VPS34-IN-1 targets the VPS34 kinase complex essential for autophagosome formation – we treated cells with Baf-A1, VPS34-IN-1, and 3 MA. The results showed that both Baf-A1 and VPS34-IN-1 significantly inhibited the degradation of OPTN[LIR]E (Figure 2(C,D), S2G). Next, OTPN[LIR]E was treated with different concentrations of 3 MA, Baf-A1, or Vps34-IN-1 for 24 h, and it was observed that the inhibitory effect of 3 MA, Baf-A1, and Vps34-IN-1 on OPTN[LIR]E was significantly concentration-dependent (Figure 2(E,F), S2H). Moreover, cells with stable OPTN[LIR]E expression were treated with 3 MA or EBSS, the latter being a well-known autophagy inducer. According to temporal gradients. Obviously, 3 MA and EBSS had a significant time-dependent effect on OPTN[LIR]E degradation regulation (Figure S2I-J). Furthermore, we monitored the fluorescence distribution in U251-OPTN[LIR]R cells following treatment with the inhibitors 3 MA, Baf-A1, and Vps34-IN-1. Notably, all three inhibitors significantly disrupted the co-localization of mRFP with lysosomes (Figure S2K-L). Thus, the degradation of OPTN[LIR]E was confirmed to be regulated through autophagy and the lysosomal pathway.

To further explore the roles of the ATG8 proteins in the degradation process of OPTN[LIR]E, we initially constructed stable cell lines that knocked out *LC3A*, *LC3B*, *LC3C*, *GABARAP*, *GABARAPL1*, or *GABARAPL2* based on the CRISPR-Cas9 system (Figure S3A). The detailed targets of the designed single gRNA are shown in Table S1. Detection of OPTN[LIR]E fluorescence levels and protein content revealed that LC3B and GABARAPL2 played more crucial roles in OPTN[LIR]E degradation than other ATG8 proteins (Figure 2(G,H), S3B-C).

The ULK complex directly participates in the initiation of autophagosome formation during selective autophagy and is essential for various types of selective autophagic processes [40]. As a critical component of the ULK complex, RB1CC1/FIP200 specifically recognizes the LIR motifs on target proteins through its claw domain located at the C terminus [41]. To further confirm that the degradation of OPTN[LIR]E is mediated by the selective autophagy pathway and to elucidate the regulatory role of RB1CC1 in this process, we established and screened stable *RB1CC1* knockout cell lines (Figure S3D). Additionally, to assess whether ATG8s lipidation is indispensable for OPTN[LIR]E degradation, we targeted ATG5, a key factor in ATG8s lipidation, and successfully achieved its knockout in cells (Figure S3D) [42]. Furthermore, we detected a strong interaction between RB1CC1 and OPTN[LIR]E, but not mOPTN[LIR]E, which was consistent with observations for LC3B (Figure S3E). Our findings suggest that the degradation of OPTN[LIR]E, rather than mOPTN[LIR]E, is mediated not only by its interaction with the conserved LIR-docking pocket of ATG8 proteins (LC3B) but also relies critically on the upstream autophagy scaffold RB1CC1. Meanwhile, no significant interaction was observed between OPTN[LIR]E and ATG5 (Figure S3E). Nevertheless, the results highlight that ATG8s lipidation is essential for the degradation of OPTN[LIR]E. Moreover, compared with several other genes, knockout of *LC3B*, *GABARAPL2*, *RB1CC1*, and *ATG5* significantly increased the intracellular fluorescence intensity and integrated density of OPTN[LIR]E cells (Figure S3F).

### Peptide-based LIR-targeting chimera degrades the target protein rapidly and efficiently

**Figure 3.**
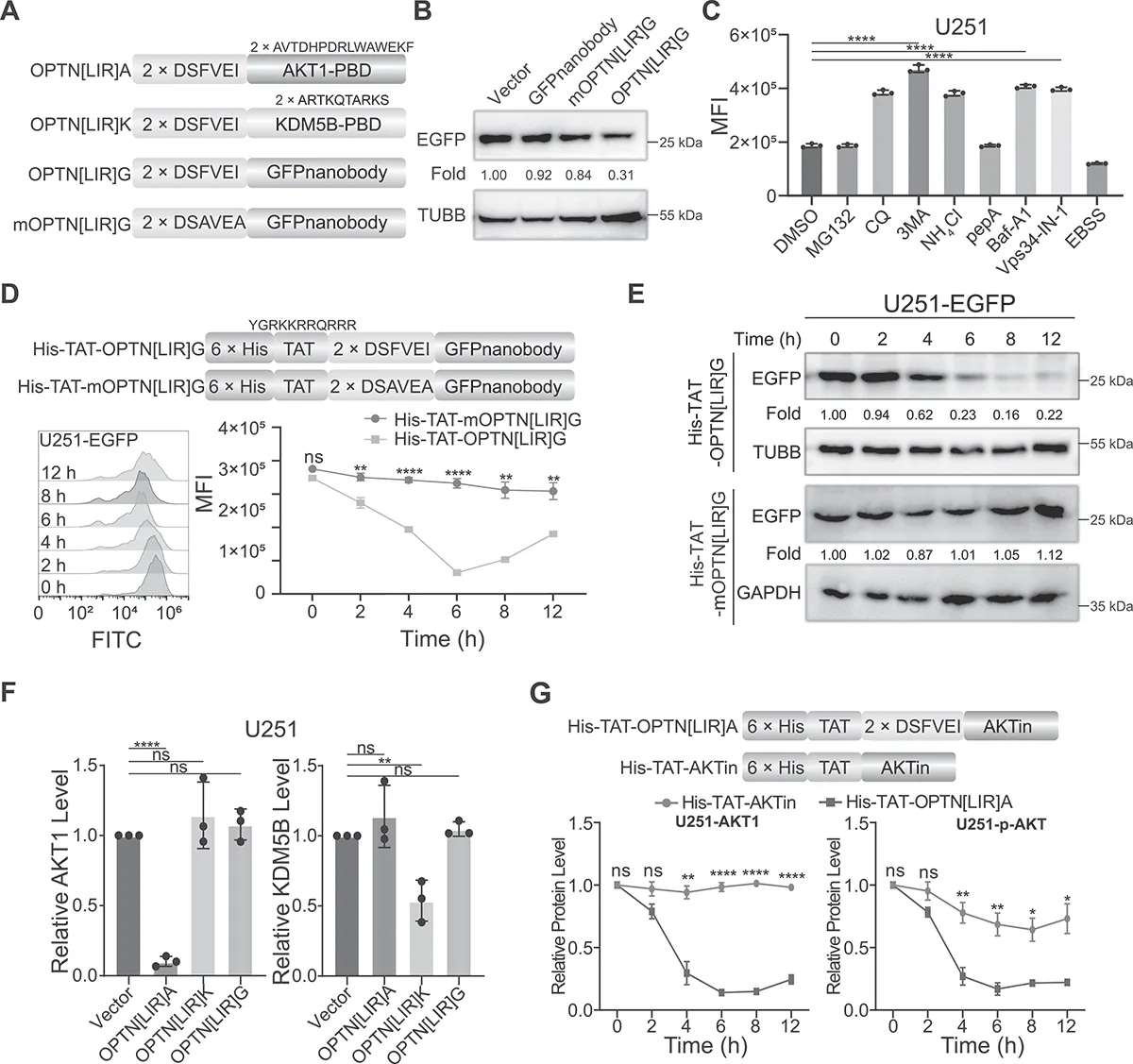

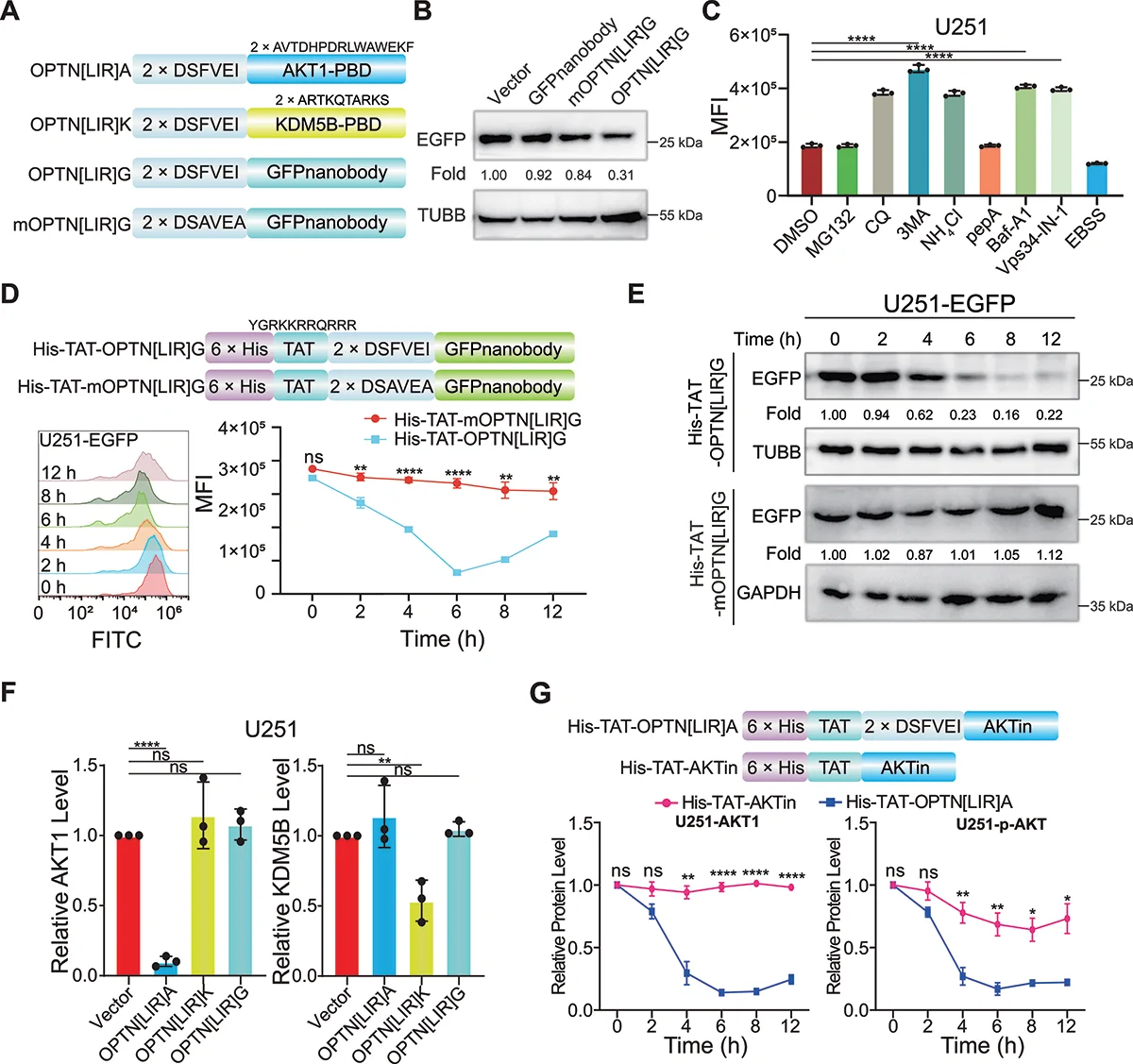
The peptide-based LIR targeting chimeras (pLIRTAC) for targeted degradation of intracellular proteins. (A) Schematic diagram of pLIRTAC recombinant sequences targeting AKT1, KDM5B, and EGFP. (B) Changes in EGFP protein levels in 293T cells treated with GFPnanobody, mOPTN[LIR]G, and OPTN[LIR]G. (C) The MFI of 293T cells stably expressing EGFP overexpressing OPTN[LIR]G after treatment with DMSO, MG132, CQ, 3 MA, NH_4_Cl, pepA, Baf-A1, VPS34-IN-1, or EBSS for 24 h. (D) Top, diagram of the recombination sequence of His-TAT-OPTN[LIR]G and His-TAT-mOPTN[LIR]G. Bottom, MFI at different time points after adding purified His-TAT-mOPTN[LIR]G (200 μM) and His-TAT-OPTN[LIR]G (200 μM) to U251 cells stably expressing EGFP. (E) EGFP protein levels at different time points in U251 cells stably expressing EGFP after adding purified His-TAT-mOPTN[LIR]G and His-TAT-OPTN[LIR]G. (F) Levels of AKT1 and KDM5B proteins in U251 cells with stable expression of OPTN[LIR]A, OPTN[LIR]K, or OPTN[LIR]G. (G) Top, diagram of the recombination sequence of His-TAT-OPTNA and His-TAT-AKTin. Bottom, AKT1 and p-AKT protein levels in U251 cells after the addition of purified His-TAT-AKTin (200 μM) and His-TAT-OPTN[LIR]A (200 μM) at different time points.

Studies on OPTN[LIR]E and OPTN[LIR]R have confirmed that proteins directly reconstituted with LIR have the opportunity to be significantly degraded via the autophagy-lysosomal pathway. Therefore, we intend to further exploit this degradation pathway to specifically degrade endogenous target proteins. Therefore, we introduced a short peptide that can specifically bind to a certain protein, and by directly recombining with LIR, LIR-targeting-chimera (pLIRTAC) was formed. Regarding the composition of pLIRTAC, in addition to requiring efficient LIR, it is also crucial to have a short peptide that can efficiently and specifically bind to the POI to degrade the target POI. To verify the effectiveness of pLIRTAC based on GFPnanobody (a nanobody that can specifically bind to GFP), we first attempted to design a pLIRTAC targeted at EGFP: OPTN-GFPnanobody (OPTN[LIR]G) to visualize its working process (Figure 3(A)) [43]. After stable expression of OPTN[LIR]G in 293T cells, the protein expression level of EGFP significantly decreased, and this reduction was even more significant in terms of fluorescence intensity levels than the GFPnanobody and mut-OPTNG (mOPTN[LIR]G) (Figure 3(B), S4A-B). To investigate whether the specific targeting degradation of EGFP by OPTN[LIR]G is dependent on the selective autophagy-lysosome pathway, we treated cells overexpressing EGFP with several inhibitors in combination with OPTN[LIR]G. 3 MA, CQ, NH_4_Cl, Baf-A1, and Vps34-IN-1 still exhibited robust inhibition of EGFP degradation, and EBSS significantly promoted the induction of EGFP degradation by OPTN[LIR]G (Figure 3(C), S4C). Additionally, the targeting-induced degradation effect of OPTN[LIR]G was significantly inhibited by knocking out *LC3B* or *GABARAPL2* in both 293T and U251 cells (Figure S4D-E). To further determine whether the intracellular localization of OPTN[LIR]G when bound to EGFP is consistent with OPTN[LIR]R, we accounted for the potential quenching of EGFP in acidic organelles such as lysosomes. Therefore, we overexpressed mOPTN[LIR]G or OPTN[LIR]G in 293T and U251 cells that stably express YFP (which can also bind to GFPnanobody) and captured magnified single-cell images. Notably, in the presence of OPTN[LIR]G, YFP exhibited significant lysosomal accumulation, similar to OPTN[LIR]R (Figure S4F-G, Correction ≥ 0.7).

Currently, disease treatment drugs mainly comprise small-molecule inhibitors and short peptides. Considering that it is difficult for short peptides to enter cells from the blood or culture media, we introduced a short peptide that can guide the peptide to cross the cell membrane: the TAT domain [44]. To obtain pLIRTACs capable of targeted protein degradation after purification and subsequent *in vitro* administration into cells, we have incorporated His tags and TAT domains into the composition of the previous pLIRTAC. Therefore, after purifying His-TAT-OPTN[LIR]G and His-TAT-mOPTN[LIR]G, we added them to the culture medium of 293T cells stably expressing EGFP. We first confirmed the successful cellular uptake of the peptides (based on His-tag flow cytometry antibodies) and observed that the degradation of EGFP was significantly dependent on the concentration of His-TAT-OPTN[LIR]G (Figure S4H-I). When His-TAT-mOPTN[LIR]G or His-TAT-OPTN[LIR]G was added to the culture medium at a concentration of 100 μM, it was observed that while His-TAT-mOPTN[LIR]G could reduce the fluorescence intensity of U251-EGFP cells, it did not decrease the intracellular levels of the EGFP protein. In contrast, His-TAT-OPTN[LIR]G significantly inhibited both the fluorescence intensity and intracellular EGFP protein levels in U251-EGFP cells (Figure 3(D,E)). Furthermore, the maximum inhibitory effect of the drug was displayed about 8 h after delivery, after which it recovered. Next, we verified whether His-TAT-OPTN[LIR]G targets EGFP degradation through the selective autophagy-lysosome pathway. As expected, even with the addition of His-TAT-OPTN[LIR]G, 3 MA could significantly reverse the degradation of EGFP, and the EGFP level in U251-EGFP cells with *LC3B*, *RB1CC1*, *ATG5*, or *GABARAPL2* knocked out was barely affected by His-TAT-OPTN[LIR]G (Figure S4J-L).

Considering the excellent performance of OPTN[LIR]G, we further utilized pLIRTAC for targeted degradation of endogenous pathogenic proteins. Studies have shown that the overexpression or abnormal activation of KDM5B or AKT1 is closely related to the occurrence and development of many tumors. Such as glioma, and targeted degradation of KDM5B or AKT1 also shows promising anti-tumor effects [45–47]. Therefore, we first designed two pLIRTACs, in which OPTN-AKTin (OPTN[LIR]A) was used to target the degradation of AKT1, and OPTN-KDM5B-binding domain (OPTN[LIR]K) was used to target the degradation of KDM5B [48,49]. When OPTN[LIR]A, OPTN[LIR]K, or OPTN[LIR]G were stably expressed in U251 cells, it was found that the targeted degradation ability of pLIRTAC was efficient and accurate by detecting the protein levels of AKT1 and KDM5B in the cells (Figure 3(F), S5A). Furthermore, molecular docking identified extensive hydrogen bonding networks in the AKT1-AKTin and KDM5B-binding domain interfaces. This finding provides the structural basis for the stable association of OPTN[LIR]K and OPTN[LIR]A with their targets, which accounts for the subsequent targeted protein degradation (Figure S5B). To further explore whether OPTN[LIR]A could affect the occurrence and development of glioma cells by knocking down the intracellular AKT, we constructed U251 cell lines that stably expressed OPTN[LIR]A or the corresponding empty expression vector. The proliferative, invasive, and migratory abilities of U251-OPTN[LIR]A were significantly inhibited. Compared with the control group and the AKTin-expressing group, U251-OPTN[LIR]A showed significant inhibition of both cell proliferation and migration (Figure S5C-E). In addition, the proliferative activity and colony-forming ability of U251-OPTN[LIR]A cells were significantly inhibited (Figure S5F-G). Finally, we attempted to design His-TAT-mut-OPTN-AKTin (His-mOPTN[LIR]A) and His-TAT-OPTN[LIR]A that could target AKT1. After entering the cells, both His-TAT-AKTin and His-TAT-OPTN[LIR]A groups showed a clear downward trend in p-AKT levels (Figure 3(G), S5H-I). However, because of the targeted degradation of intracellular AKT1 by His-TAT-OPTN[LIR]A, only the His-TAT-OPTN[LIR]A group could effectively reduce the levels of both AKT1 and p-AKT at the same time (Figure 3(G), S5H). The inhibitory effect peaked at around 8 h post-treatment. Additionally, 3 MA treatment abolished OPTN[LIR]A-mediated degradation of AKT1 (Figure S5J). In conclusion, pLIRTAC could efficiently and accurately target the degradation of endogenous proteins through the autophagy-lysosome pathway *in vitro*, and further affect the biological behavior of cells.

### Glioma development can be inhibited by pLIRTAC through targeted degradation of AKT1

**Figure 4.**
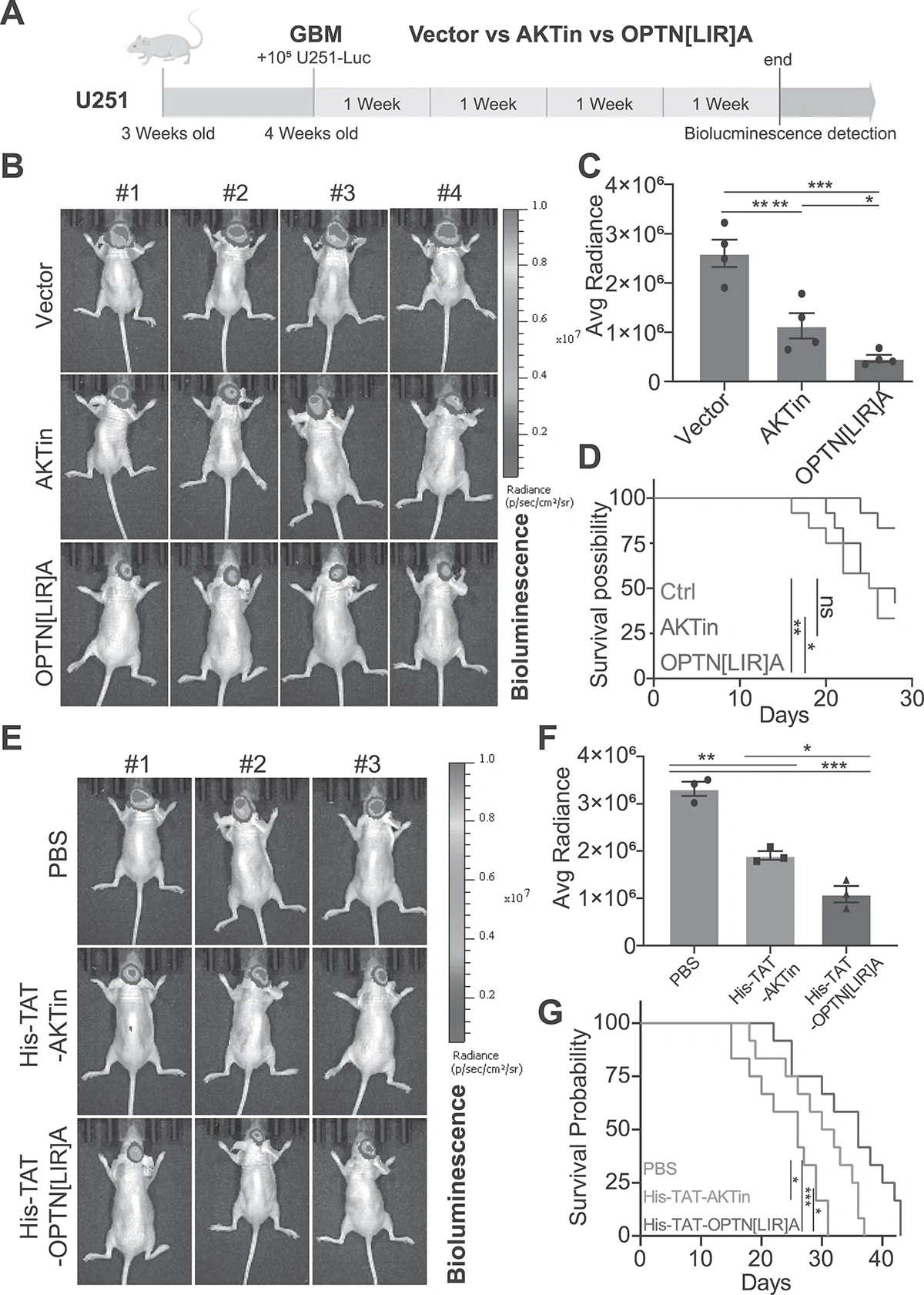

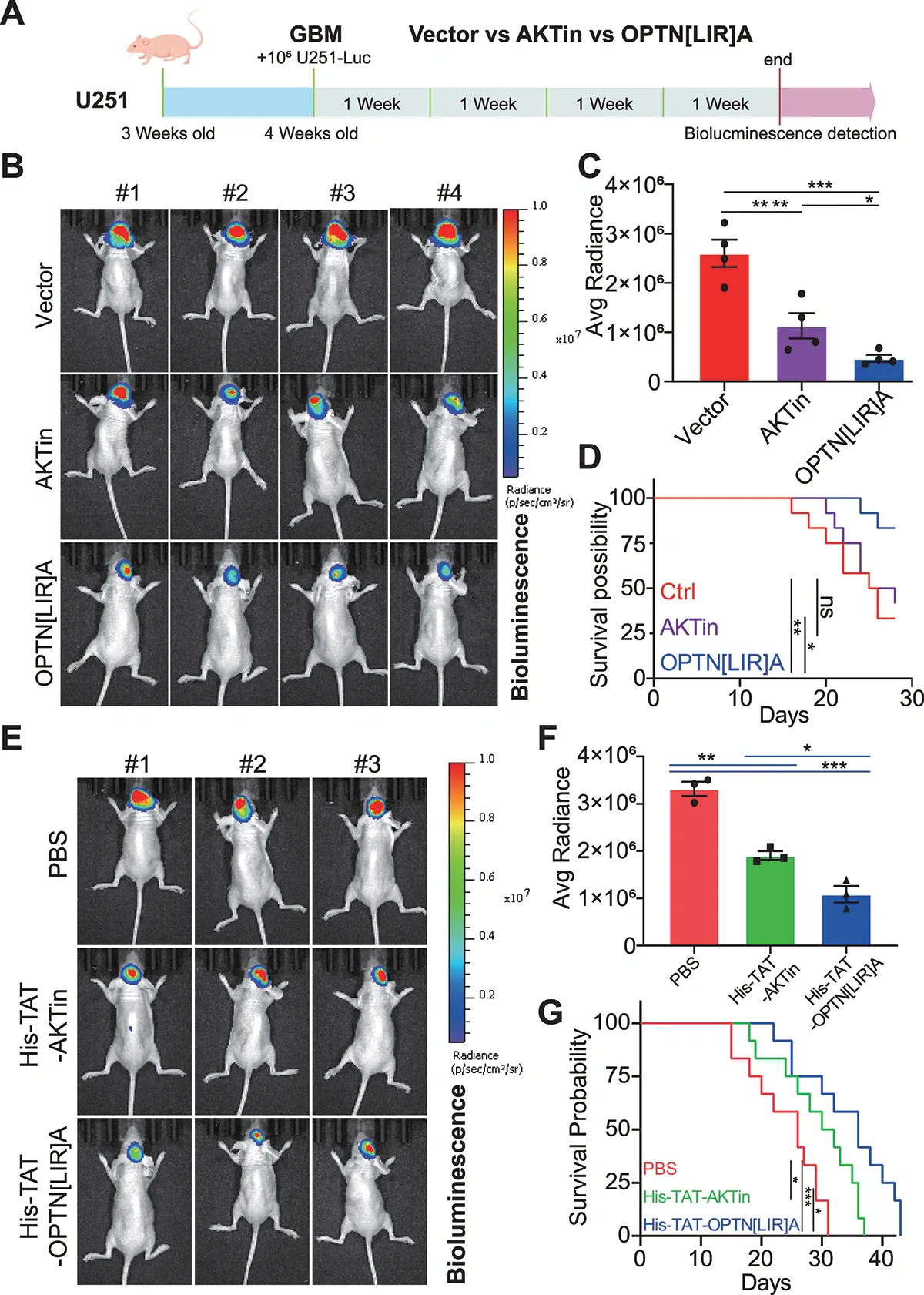
pLIRTAC can inhibit the development of glioma by knocking down AKT1 *in vivo*. (A) Experimental timeline of the U251 GBM model. (B) Live imaging of glioma mice with stable expression of vector, AKTin, or OPTN[LIR]A at the end of the experiment. And the average bioluminescence intensity (C) and survival probability (D) of each group (12 mice per group). (E) Bioluminescence photos of GBM mice given PBS, His-TAT-AKTin, or His-TAT-OPTN[LIR]A on day 28 of the establishment of the GBM mouse model. And the average bioluminescence intensity (F) and survival rate (G) of each group.

**Figure 5.**
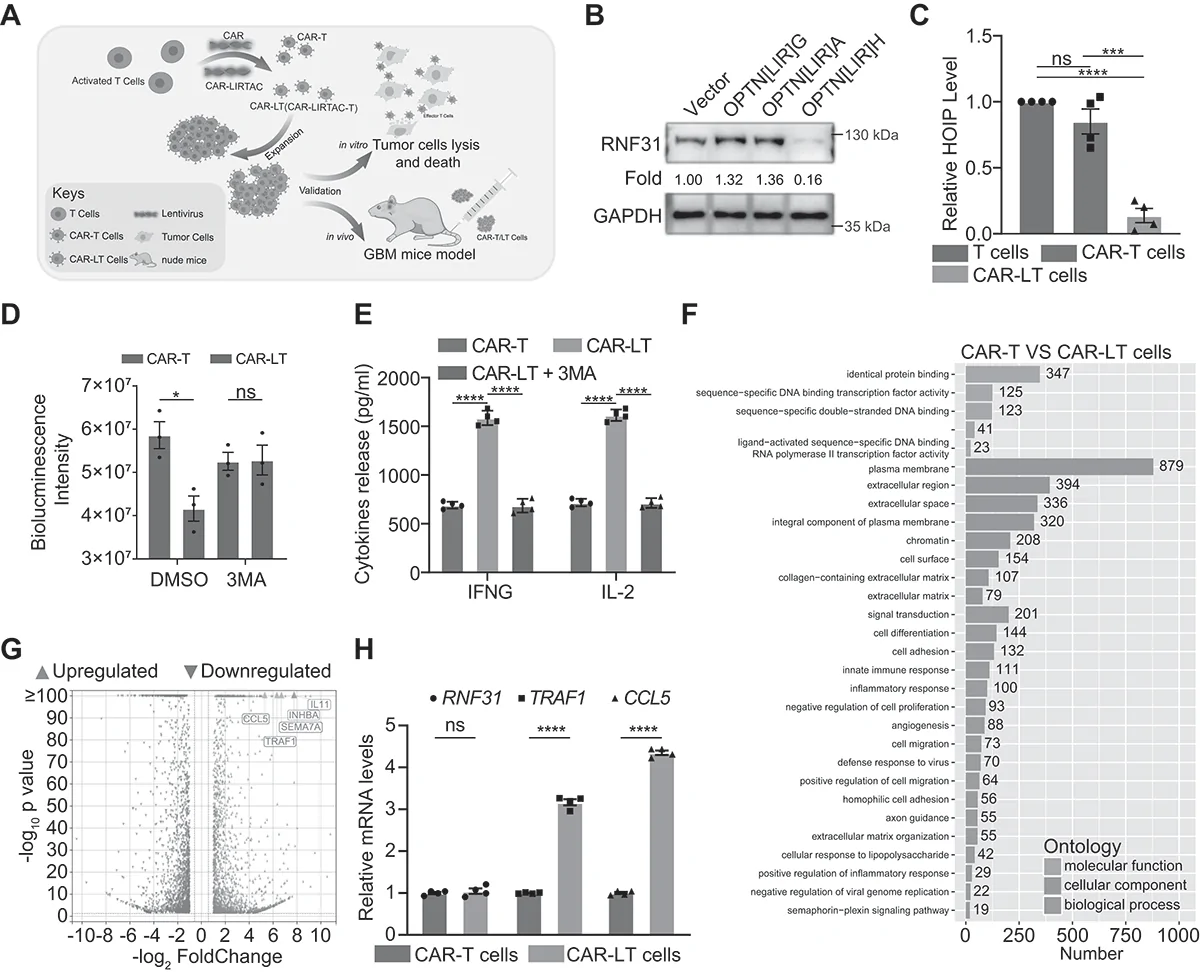

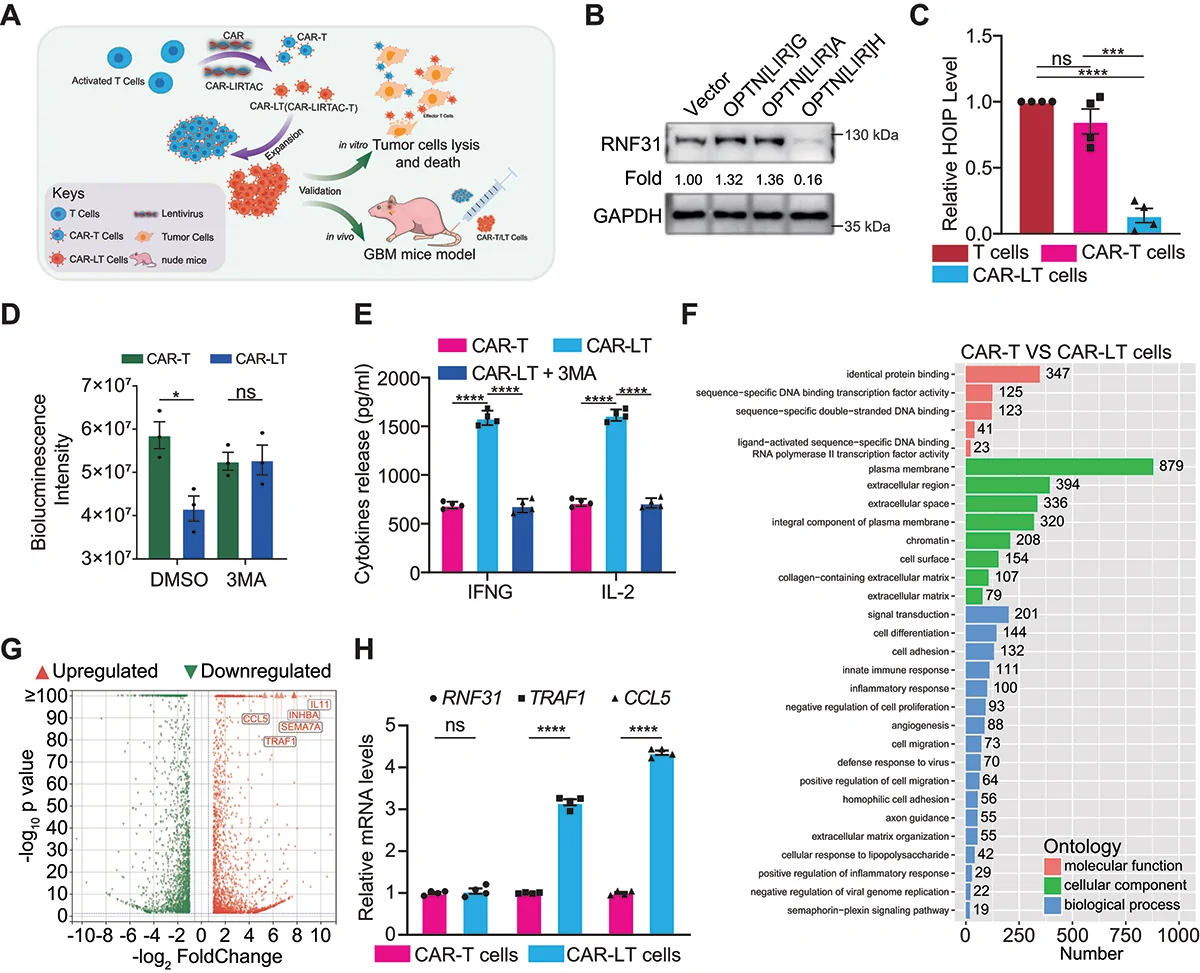
OPTN[LIR]H can significantly improve the persistence of CAR-T cells. (A) Generation of CAR-T/CAR-LT cells and schematic diagram of *in vitro* and *in vivo* anti-tumor experimental validation. (B) The level of RNF31 protein was different after different pLIRTACs were expressed in T cells. (C) RNF31 protein levels in t, CAR-T, and CAR-LT cells. (D) Bioluminescence intensity of CAR-T/CAR-LT cocultured with U251-luc cells after 48 h treatment with DMSO or 3 MA. (E) Cytokine release levels detected in CAR-T/CAR-LT/CAR-LT and 3 MA cell media. (F) KEGG analysis shows significant differences in pathway enrichment in CAR-LT cells and CAR-T cells. (G) The volcano plot shows the genes that are upregulated or downregulated in CAR-LT cells relative to CAR-T cells. (H) Differences in mRNA expression of *RNF31*, *TRAF1*, and *CCL5* between CAR-T and CAR-LT cells.

AKT1 has been recognized as an attractive target for cancer therapy, and the carcinogenicity of AKT is mainly because of its promotion of cell proliferation, invasion, and activation of anti-apoptotic signals [45,50]. With the help of pLIRTAC, we have successfully achieved targeted degradation of AKT1 and p-AKT and effectively suppressed malignant behaviors such as proliferation and invasion of glioma cells *in vitro*. However, further validation of pLIRTAC’s effectiveness *in vivo* is necessary. Therefore, we first stably overexpressed luciferase in U251 cells, and then stably overexpressed the AKTin or OPTN[LIR]A, respectively. These cells were used to construct intracranial glioma models in 4-week-old male nude mice and were divided into three groups. The flow chart of the *in vivo* experiments of these nude mice is presented in Figure 4(A). The weight changes of each nude mouse were closely monitored after tumor formation, and *in vitro* imaging was performed on nude mice on the 28th day after tumor formation, when the experiment was terminated. The weight of mice in the OPTN[LIR]A group was always higher than that in other groups after tumor formation (Figure S5N). By evaluating the average bioluminescence signal of each group of mice, we found that glioma was significantly inhibited in the AKTin group, while the inhibition effect was further improved in the OPTN[LIR]A group (Figure 4(B,C)). In addition, the overall survival of the OPTN[LIR]A group was significantly longer than that of the control group and AKTin group, but there was no significant difference between the control group and AKTin group (Figure 4(D)). Meanwhile, IHC staining of mouse brain tissues demonstrated that GBM tumors from the OPTN[LIR]A-treated group exhibited markedly reduced levels of both phosphorylated and total AKT1 relative to the other groups (Figure 5(O)).

To further examine the efficacy of His-TAT-pLIRTAC *in vivo*, intracranial glioma models were constructed using the U251-luciferase cells described above, using a similar principle, and mice were divided into three groups, each of which received intrathecal injections of PBS, His-TAT-AKTin, or His-TAT-OPTN[LIR]A. The specific time points of each treatment in this batch of nude mice are shown in Figure S6A. We found that the average weight of nude mice in the His-TAT-OPTN[LIR]A group was still higher than that in the other two groups (Figure S6B). Although His-TAT-OPTN[LIR]A could still significantly reduce the intracranial bioluminescence signal of glioma mice, and its overall survival time was also significantly extended, the anti-tumor effect of Intrathecal injection of drugs on glioma mice was slightly worse than the stable expression of OPTN[LIR]A (Figure 4(E-G)). This may be related to the rapid drug metabolism and weak cell targeting of His-TAT-pLIRTAC.

### Degradation of RNF31/HOIP by pLIRTAC inhibits T cell exhaustion

CAR-T cell therapy has demonstrated considerable and reliable efficacy in the treatment of hematological malignancies and is gradually being tested and validated in the treatment of solid tumors [26,51]. It is noteworthy that in the process of tumor evasion from CAR-T cell killing, many key genes have been identified as critical players [52–54]. Moreover, it has been demonstrated that the suppression of certain genes within tumor cells or immune cells can significantly enhance the anti-tumor efficacy of immune cells [55–57]. Interestingly, many modifications to CAR-T cells were aimed at improving their persistence and stability, thereby enhancing their anti-tumor efficacy, and this strategy has achieved remarkable success. Therefore, combining gene therapy with CAR-T cell anti-tumor therapy is a very promising strategy, and there is an urgent need to explore more potential high-efficiency targets.

RNF31, a member of the RBR E3 ligase, has been found to be overexpressed in a variety of cancers and has been shown to be widely involved in the malignant progression of tumor cells [58–61]. More importantly, upregulation of RNF31 can significantly inhibit tumor cell apoptosis and reduce the sensitivity of a variety of cancer cells to immune cell therapy and chemotherapy [62–64]. Several studies have confirmed that downregulating RNF31 in tumor cells can significantly increase the sensitivity of tumor cells to immune cell therapy [63,65].

Although studies have shown that the expression level of RNF31 is significantly correlated with the lifespan of T cells, there is a lack of direct evidence that RNF31 protein levels affect the anti-tumor effects of CAR-T cells [56]. Therefore, based on the successful experience of pLIRTAC, we designed OPTN-RNF31 single-domain antibody (OPTN[LIR]H) that can target and degrade RNF31 with the help of the reported single-domain antibody targeting RNF31 [66]. To generate CAR-T cells stably expressing OPTN[LIR]H, we first purified PBMCs isolated from the whole blood of healthy volunteers. T cells were activated and expanded by binding to CD3/CD28 beads and then cultured in a medium containing IL2 (Figure S6C). Figure 5(A) briefly explains how activated T cells were used to construct CAR-T/CAR-pLIRTAC-T (CAR-LT) cells and the anti-tumor efficacy verification process *in vitro* and *in vivo*.

We first verified the specificity and targeted degradation efficiency of OPTN[LIR]H, and the results showed that compared with HSDA (RNF31 single-domain antibody), OPTN[LIR]H could accurately and efficiently regulate the protein level of RNF31 in T cells without affecting the RNF31 mRNA level (Figure 5(B), S6D). Unlike the traditional short-hairpin RNA knockdown target protein or knockouts of the CRISPR Cas9 system, pLIRTAC does not require an additional lentiviral vector; it can be attached to the C terminus of the CAR sequence with the help of the P2A peptides that can significantly reduce the screening cycle required for lentiviral infection and the associated damage to the genome. Therefore, we successfully constructed CAR-T and CAR-LT cells and found that the RNF31 protein level in CAR-LT cells was significantly lower than that in the CAR-T cells, and the RNF31 protein level in CAR-LT cells significantly increased following 3 MA treatment (Figure 5(C), S6E). Interestingly, the tumor cell killing effect of CAR-LT cells can also be downregulated by 3 MA (Figure 5(D)). To investigate whether depletion-related indicators of CAR-LT cells changed, we examined cytokines released by CAR-T and CAR-LT cells. Our results showed that the IFNG/IFN-γ and IL2 release of CAR-LT cells were significantly higher than that of CAR-T cells (Figure 5(E)). However, this enhanced cytokine production in CAR-LT cells was significantly abolished by 3 MA co-treatment (Figure 5(E)). This likely indicates that OPTN[LIR]H can effectively inhibit the exhaustion status of CAR-T cells [67,68].

### pLIRTAC increases anti-tumor efficacy by enhancing CAR-T cell persistence

**Figure 6.**
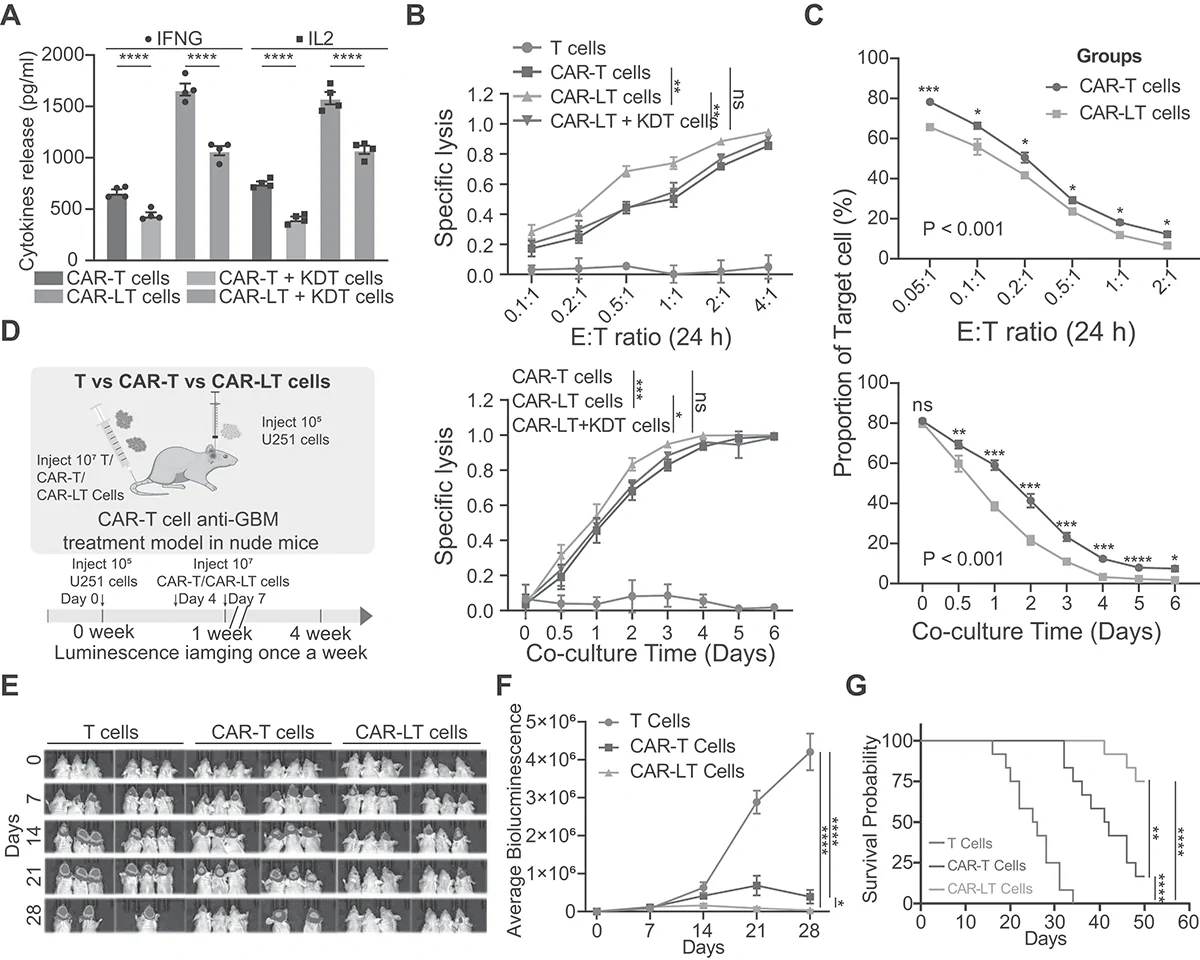

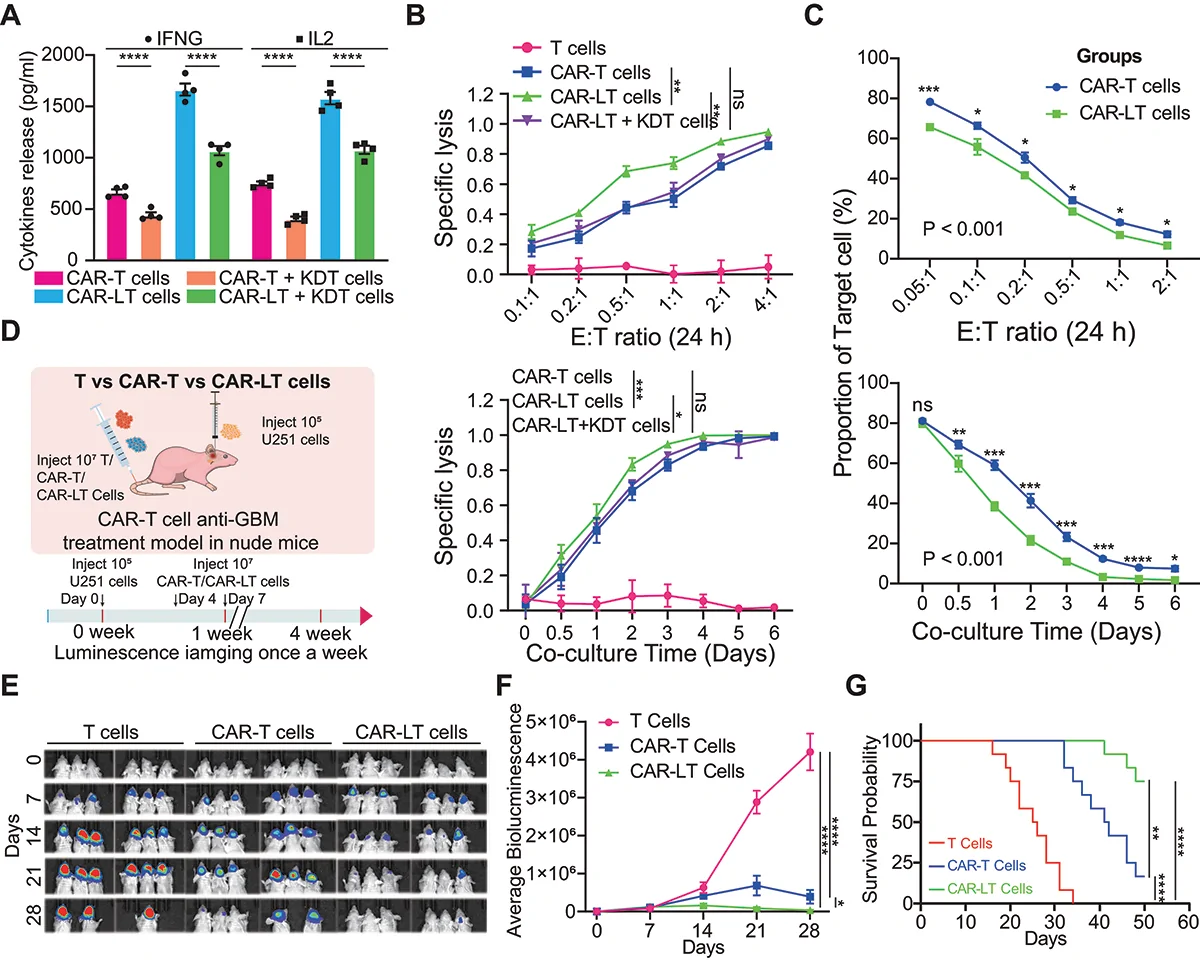
pLIRTAC can significantly improve the efficacy of CAR-T cell antitumor therapy *in vitro* and *in vivo*. (A) Cytokine release levels detected in CAR-T/CAR-LT/CAR-T + KDT/CAR-LT + KDT cell media. (B) Top, tumor target cell lysis efficiency in T/CAR-T/CAR-LT/CAR-T + KDT groups at different E:T ratios. Bottom, the distribution of target U251 cells after co-culture of CAR-T or CAR-LT cells with target cells at different time points when E:T ratio was 0.2:1. (C) Top, the proportion of target cells after 24-h co-culture of CAR-T or CAR-LT cells with target cells at different E:T ratios. Bottom, the proportion of target cells after co-culture of CAR-T or CAR-LT cells with target cells at different time points when E:T ratio was 0.2:1. (D) Schematic diagram of an animal experimental. (E) Bioluminescence photos of GBM nude mice using T/CAR-T/CAR-LT cells for anti-tumor experiments *in vivo*. And the average bioluminescence intensity (F) and survival rate (G) of each group (12 mice per group).

To elucidate the mechanism by which RNF31 regulates CAR-T cell persistence, we collected CAR-T and CAR-LT cells for RNA sequencing analysis. Interestingly, it can be seen that signaling pathways such as “cytokine activity” and “TNF signaling pathway” are significantly enriched in CAR-LT cells (Figure 5(F), S6F). In addition, we found that apoptosis-related proteins TRAF1 and CCL5 were significantly upregulated in CAR-LT cells (Figure 5(G)) [69–71]. We then verified the expression levels of these two genes in CAR-LT cells, and the results suggested that the reduction of RNF31 at the protein level could significantly increase the transcription levels of TRAF1 and CCL5 (Figure 5(H)). CCL5 has been reported to promote T cell survival and anti-apoptosis by activating some signaling pathways, such as the PI3K signaling pathway, and even promote T cell immune activation and persistence, so we did not conduct further research [72]. We initially observed that the RNF31 protein was downregulated in CAR-LT cells, accompanied by a significant upregulation of TRAF1 protein (Figure S6G). Furthermore, overexpression of the TRAF1 in CAR-T cells markedly reduced the proportion of apoptotic cells (Figure S6H). To further verify whether RNF31 can enhance T cell persistence by upregulating the expression level of TRAF1, we knocked down the expression level of the *TRAF1* gene in CAR-LT cells (CAR-LT-KDT) and found that the levels of IFNG and IL2 were lower than those in CAR-LT cells (Figure 6(A)). Furthermore, we found that 3 MA significantly downregulated the TRAF1 protein level in CAR-LT cells and consequently attenuated their persistent tumor-killing capacity (Figure S6I-J).

Next, we validated the anti-tumor cell efficacy of CAR-LT cells *in vitro*. In the co-culture of effector T cells and target cells (U251-CD19^+^ luciferase), CAR-LT cells showed stronger tumor cell lysis than CAR-T cells, and the overall anti-tumor effect was significantly improved with the increase in the proportion of CAR-LT cells, while the downregulation of TRAF1 weakened the anti-tumor effect of CAR-LT cells (Figure 6(B)). Similarly, U251-CD19^+^ EGFP cells were tested for real-time target cell ratios after co-culture with CAR-T or CAR-LT cells. Surprisingly, CAR-LT showed more robust anti-tumor therapeutic efficacy than CAR-T cells (Figure 6(C)). To further validate the efficacy of the constructed CAR-LT cells for anti-glioma therapy *in vivo*, we established intracranial glioma models based on U251-CD19^+^ luciferase cells using the aforementioned method. The process of CAR-LT treatment for glioma was illustrated in Figure 6(D). After tumor formation, the mice were equally divided into four groups and intravenously injected with PBS, T-cells, CAR-T cells, or CAR-LT cells on the day 3 and 10, respectively, after tumor formation. To visually demonstrate the anti-tumor effects of CAR-T cells, we dynamically monitored the survival status of the mice in each group. Additionally, six randomly selected mice in each group were continuously tested for intracranial bioluminescence intensity once a week (Figure 6(E)). According to the average bioluminescence intensity, both CAR-T cells and CAR-LT cells showed excellent anti-tumor effects, and OPTN[LIR]H supplementation could still significantly improve the anti-tumor effect of CAR-T cells and further prolong the overall survival of mice with glioma (Figure 6(F,G)).

The above results indicated that the combination of OPTN[LIR]H and CAR could significantly enhance the persistence of CAR-T cells by reducing the endogenous RNF31 protein level of T cells, thus producing a more excellent anti-tumor effect.

## Discussion

Despite its short history since inception, TPD has rapidly developed and demonstrated an extremely broad application potential [73]. As an alternative to the widely recognized traditional small-molecule inhibitors, the current TPD approach has spawned various technologies, of which PROTAC and molecular glue technology (both based on the ubiquitin-proteasome system) are more mature [1]. With increasing understanding of the lysosomal degradation pathway, various TPD approaches targeting the degradation of intracellular and extracellular proteins, or even damaged organelles, have been gradually developed [74–76]. However, TPD technology utilizing the selective autophagy-lysosome pathway for validation in anti-tumor therapy still needs further exploration. In this context, we developed the pLIRTAC technology to efficiently and specifically degrade target proteins through the autophagy-lysosome degradation pathway, thereby demonstrating its outstanding anti-tumor capabilities. Notably, pLIRTAC can be combined with CAR to further enhance the anti-tumor therapeutic effects of CAR-T cells.

T cell exhaustion is a major limiting factor in the current CAR-T cell anti-tumor immunotherapy [77]. Several genes influencing T cell exhaustion have been identified, and regulating these genes has been shown to potentially enhance T cell responses in anti-tumor therapy [26,78]. However, simultaneously incorporating CAR while regulating these proteins within T cells remains a challenge. In our innovative approach, we incorporated pLIRTAC and CAR sequences into the same lentivirus to enable modified T cells to specifically recognize target tumor cells, while reducing proteins that promote T cell exhaustion and further enhancing the enduring anti-tumor capabilities of CAR-T cells.

In our research, we initially selected different LIR motifs of various SARs and recombinantly expressed them with EGFP or mRFP. Surprisingly, proteins recombinantly expressing the LIR motif from the OPTN protein exhibited exceptional degradation efficiency, while SQSTM1, a classic SAR, did not yield any significant results. Furthermore, we verified that OPTN[LIR]E and OPTN[LIR]R were degraded via the autophagy pathway in the lysosome and were significantly regulated by LC3B and GABARAPL2 proteins. This phenomenon may be related to the critical roles and unique functions of LC3B and GABARAPL2 in the autophagic process: Knockdown of LC3 or GABARAP subfamily proteins results in distinct autophagic impairment phenotypes, whereas re-expression of LC3B or GABARAPL2 is sufficient to restore autophagy. LC3B rescues phagophore elongation, while GABARAPL2 mediates vesicle closure [79,80]. To rapidly degrade pathogenic proteins, we introduced pLIRTAC to specifically target and degrade AKT1 and KDM5B and achieved significant results. The rapid and reversible degradation of the target protein could also be achieved when pLIRTACs were purified and delivered *in vitro*, which greatly expanded the developmental potential of pLIRTAC. This also means that pLIRTAC can be directly used after being expressed and purified by bacteria, and the treatment strategies can be optimized by adjusting the medication time and concentration of synthetic drugs. In animal experiments, the stable expression of OPTN[LIR]A – a pLIRTAC that has been shown to significantly degrade intracellular AKT and p-AKT levels *in vitro*—can effectively inhibit the occurrence and development of glioma tissues and significantly improve the prognosis of glioma mice. In subsequent in-depth studies, we also tried to apply the effective drug delivery system verified in this study in mice, and His-TAT-OPTN[LIR]A showed superior efficacy compared to His-TAT-AKTin. However, because of the weak targeting of His-TAT-OPTN[LIR]A, it may not be precisely targeted to tumor cells and may induce a decrease in AKT levels in many normal tissue cells. In addition, given the short metabolic cycle of drug delivery, the stability of the treatment effect after AKT reduction in tumor cells is significantly weaker than that in glioma tissues with persistent OPTN[LIR]A expression. Therefore, we intend to improve the deliverable pLIRTAC drug in subsequent studies to enhance its targeting and stability. Moreover, we applied pLIRTAC combined with CAR to the anti-tumor immunotherapy of CAR-T cells. The pLIRTAC sequence only needed to be expressed at the C terminal of the CAR sequence so that both could be transferred into T cells and expressed at the same time. This attempt significantly improved the efficacy of the anti-tumor effect in GBM-bearing mice. Based on the above conclusions, we can speculate that pLIRTAC can be used to target the degradation of multiple exhaustion-related proteins of various immune cells or inhibit infiltration-related proteins of some immune cells to enhance their anti-tumor therapeutic effect. In future experiments, we plan to verify whether multiple pLIRTACs simultaneously targeting the degradation of multiple exhaustion-related proteins can further improve the anti-tumor ability of CAR-T cells.

Different from TPD technology based on the ubiquitin-proteasome system, pLIRTAC mainly relies on the autophagy-lysosome pathway to achieve efficient degradation of target proteins. pLIRTAC contains an LIR motif that recruits the “cargo” to autophagosomes and a short peptide that can specifically target and bind to the POI. If purified pLIRTAC drugs are needed for delivery into cells, tags that are suitable for purification and transmembrane domains need to be added to pLIRTAC. In this study, pLIRTAC was noted to be precise and efficient and could rapidly degrade target proteins. It also showed dose- and time-dependent characteristics. And pLIRTAC can specifically knock down endogenous proteins while barely affecting the genetic material in the cell nucleus, providing a valuable theoretical basis for the effective application and promotion of pLIRTAC.

Compared to these established and highly promising TPD strategies – AUTAC, which selectively degrades intracellular proteins, organelles, and lipids via ubiquitin-mediated autophagy; ATTEC, which directly tethers aggregated or dysfunctional proteins to autophagosomes for efficient clearance with low toxicity; and LYTAC, which enables the lysosomal degradation of extracellular and membrane proteins with high specificity using engineered antibodies – the significant advantages of pLIRTAC lie in its ease of engineering, strong biocompatibility, and seamless integration with CAR-T cell therapy for enhanced therapeutic potential [81–83]. While pLIRTAC shares similar mechanisms and structural elements with LIR-based approaches targeting protein aggregates and organelles, it potentially outperforms in terms of size, versatility, and modifiability, offering broader applications and greater adaptability [84,85]. However, like all TPD technologies, some inevitable limitations need to be addressed. The components of our developed pLIRTAC are mainly short peptides, making it difficult to guarantee intracellular stability. Furthermore, finding and screening short peptides that can efficiently bind to the target protein is not easy. Because of the ease of screening and the excellent ability to bind specifically to target proteins, small-molecule drugs are currently mainly used as small-molecule inhibitors in similar research. Therefore, continuing to explore pLIRTAC based on small-molecule compounds is our next research direction. We also found that pLIRTAC is currently mainly suitable for degrading cytoplasmic proteins, and the efficiency of degrading target proteins located in other subcellular compartments, such as the cell membrane or nucleus, needs further improvement. Additionally, the direct application of pLIRTAC in the treatment of pathogenic proteins may lead to unforeseen side effects, such as degrading target proteins in non-target cells and causing a series of physiological changes in normal cells. However, we believe that this limitation will be significantly improved when combined with CAR-T therapy.

In summary, we have presented a peptide-based pLIRTAC technology that can precisely, efficiently, and rapidly reduce endogenous proteins at the protein level. pLIRTAC has shown excellent performance, both *in vitro* and *in vivo*. It can directly target the degradation of carcinogenic proteins in tumor cells to achieve anti-tumor purposes and can also be used to target the degradation of exhaustion-related proteins in T cells to improve the anti-tumor therapeutic efficacy of CAR-T cells. As a significant supplement to TPD technology, pLIRTAC not only serves as a powerful tool for scientific research but also holds promising clinical translational prospects.

## Materials and methods

### Antibodies and chemicals

Antibodies against human TUBB/beta-tubulin (10068–1-AP), GAPDH (60004–1-Ig), GFP (50430–2-AP and 66,002–1-Ig), AKT1 (10176–2-AP and 60,203–2-Ig), p-AKT (66444–1-Ig), Flag (20543–1-AP), LC3A (18722–1-AP), LC3C (18726–1-AP), GABARAP (18723–1-AP), GABARAPL1 (66458–1-Ig), RNF31/HOIP (30797–1-AP), Cas9 (26758–1-AP), RB1CC1/FIP200 (68564–1-1 g), MKI67/Ki-67 (27309–1-AP), and ATG5 (66744–1-lg) were purchased from Proteintech. LC3B monoclonal antibody was purchased from Cell Signaling Technology (83506), GABARAPL2 monoclonal antibody was purchased from Abclonal (A9585). Antibodies against human KDM5B (ab181089) and TRAF1 (ab300075) were obtained from Abcam.

3-Methyladenine (3 MA, HY-19312), chloroquine (CQ, HY-17589A), pepstatin A (pepA, HY-P0018), ammonium chloride (NH_4_Cl, HY-Y1269C), Baf-A1 (HY-100558), VPS34-IN-1 (HY-12795), and MG132 (HY-13259) were purchased from MedChemExpress, and dimethyl sulfoxide (DMSO, D915512) and D-luciferin potassium salt (D812647) were procured from Macklin. LysoTracker Green (C1047S) was purchased from Beyotime Biotechnology. Crystal violet (C8470), Coomassie Brilliant Blue G-250 (C8420), Hoechst 33,342 (C0031), Earle’s balanced salt solution (EBSS, H2045), and 4% paraformaldehyde (P1110) were purchased from Solarbio.

### Construction of plasmids and mutagenesis

The pLV-eGFP vector, used for overexpression, was gifted by Pantelis Tsoulfas (Addgene, 36,083). After digestion with XbaI and AgeI restriction enzymes, T4 DNA ligase (New England Biolabs, M0202V) was utilized for ligation with various nucleotide sequences of LIR motifs, including *SQSTM1*, *OPTN*, *STBD1*, *CALCOCO2*, and *NBR1*. Fragment synthesis and sequencing verification were performed by Sangon. Following the addition of monomeric red fluorescent protein (mRFP) and P2A, a self-cleaving 2A peptide, fragments at the N terminus of enhanced green fluorescent protein (EGFP) in the vector, similar methods were used to construct wild-type mRFP (WT-mRFP) and LIR (OPTN)-mRFP (OPTN[LIR]R). To construct a more reliable mutant control, we designed mutation primers to introduce the DSFVEI to DSAVEA substitution. The mutation was performed using the Novozymes point mutation kit (Vazyme, C112), following the manufacturer’s instructions. For the pLIRTAC targeting endogenous proteins, EcoRI and XbaI double enzyme digestion was performed on the vector, followed by ligation with directly synthesized nucleotide fragment annealing products using T4 DNA ligase. The CAR or CAR-pLIRTAC (CAR-L) sequence constructs for transfection into T cells were synthesized by the company directly in the empty pCDH-EF1 vector (gifted by Kazuhiro Oka; Addgene, 72,266). All primers were synthesized with the assistance of Sangon.

### Cell culture and treatment

The HEK 293T, U87MG, and U251MG cells researched in the study were purchased from ATCC (CRL-3216, HTB-14, and THB-17) and cultured in Dulbecco’s Modified Eagle’s Medium (DMEM; Thermo Fisher Scientific, 11,966,025) supplemented with 10% fetal bovine serum (FBS). T cells were cultured in RPMI 1640 medium (Thermo Fisher Scientific, 11,875,093) containing 10 ng/ml IL2 and 10% FBS. All cells were maintained in a cell culture incubator at 37°C with 5% CO_2_. Lipofectamine 3000 (Invitrogen, L3000015) was utilized for the transfection of plasmids when the cells reached 80% confluence in culture dishes or plates.

For lentiviral infection, concentrated lentivirus prepared using a lentivirus concentration kit (BIOMIGA, V2001) was added to DMEM or RPMI 1640 medium after titer determination, filtered through a 0.22-μm membrane, and used to infect cells. The medium was then replaced with complete growth medium 24 h later, and stable transduced cells were obtained through drug screening. In order to obtain a monoclonal stable cell line, we diluted the cells infected with sgRNA lentivirus to ≤2 cell/ml, cultured and expanded them in a 48-well plate, and gradually transferred the expanded monoclonal subpopulation to a 6-well plate. We continued to apply screening pressure (puromycin) and performed western blotting detection on the extracted proteins to select the cell line with the highest knockout efficiency for subsequent experiments.

### Western blotting

For cells that were centrifuged after digestion, the cell pellet was resuspended in RIPA lysis buffer (containing protease and phosphatase inhibitors from Cell Signaling Technology, 9806). After lysed and centrifuging, the supernatant was mixed with loading buffer (Solarbio, P1040) and heated at 100°C for 10 min. The above prepared samples were loaded onto 10% or 12% SDS-PA GE gels at 25 μg per lane, separated, and then transferred to PVDF membranes. For proteins obtained from bacterial sonication, direct Coomassie Brilliant Blue staining was used to identify sample positions and purity. For cell lysates, after blocking with 5% bovine serum albumin (BSA; Solarbio, A8010), the membranes were incubated with corresponding primary antibodies on a horizontal shaker overnight at 4°C. Subsequently, corresponding secondary antibodies were used to incubate the membrane at room temperature (RT) for 1–2 h. Then the membranes were immersed in ECL ultra-sensitive chemiluminescent substrate for a few seconds and imaged in the Multifunctional chemiluminescence imaging system (Tanon, China). Finally, the band grayscale values were quantitatively analyzed using ImageJ software (1.53t).

### Recombination protein expression and purification

Using molecular cloning techniques, His-TAT-OPTN[LIR]A and His-TAT-mOPTN[LIR]A were cloned into the pET28-MHL vector (pET28; Addgene, 26,096) and transformed into *Escherichia coli* BL21 (DE3). The transformed bacteria were incubated on agar plates containing 50 μg/ml kanamycin. Monoclonal colonies were selected and amplified in Luria-Bertani (LB) medium. The bacteria were further cultured with 0.5 mM IPTG (Beyotime, ST098) at 20°C for 16–18 h when the OD 600 nm reached 0.6–0.8, followed by bacterial collection through centrifugation after sonication. After ultrasonic treatment and filtration, the bacterial lysate was placed in the AKTA start protein purification system (Cytiva, USA) for purification. Purification of the His-tagged peptides strictly followed the manufacturer’s instructions (HisTrap HP, Cytiva). Briefly, the nickel column was equilibrated before loading the prepared bacterial extract. The column was then washed before applying the elution buffer. The purity of the purified peptides was determined using Coomassie brilliant blue staining, and the elution conditions were optimized. The bicinchoninic acid (BCA) assay was used to determine peptide concentration. The purified His-tagged protein was prepared in serum-free medium to the desired concentration and then directly added to serum-free medium containing cells.

### Luciferase-based cytolysis assay

To assess the anti-tumor capability of T cells *in vitro*, a luciferase-based cytolysis assay was performed. First, a luciferase-CD19-EGFP sequence was introduced into U251 cells (U251-CD19). Targeted U251-CD19 cells (T) were diluted to a density of 1 × 10^5^ and evenly distributed in opaque 96-well plates at 100 μl per well. Effector T cells (E) were added at specific ratios (100 μl per well) and co-cultured for the desired duration. Subsequently, 10 μl of stable Glo luciferase substrate (Promega, G7941) was added to the lysate of co-cultured cells and incubated at RT for 5 min. Similarly, to determine the cell lysis efficiency at different time points under a fixed E:T ratio, the co-culture system was cultured until a specific time point for lysis, followed by the addition of 10 μl stable Glo luciferase substrate. The luminescence intensity was recorded using a multiscan spectrum (PerkinElmer, USA). The specific lysis percentage was calculated as follows: %cytotoxicity = 100% × (1-[RLU of lysate of co-cultured cells])/(RLU of target U251-CD19 cells alone).

### Generation of CAR-T/CAR-L-T cells

Peripheral blood mononuclear cells (PBMC) of healthy volunteers were collected from the Second Affiliated Hospital of Nanchang University (Nanchang, China) with approval from their ethics committee. For primary T cell isolation and purification, peripheral whole blood provided by healthy volunteers was processed following the manufacturer’s instructions using human lymphocyte separation medium (Solarbio, China) and human CD3^+^ T cell sorting kit (Beaver, 71,003). Anti-CD3/CD28 antibody conjugated magnetic beads (Beaver, 70,910) and IL2 were used to stimulate and activate primary T cells, respectively, to promote their expansion. After 15 days of expansion, the beads and the expanded T cells were ready for subsequent experiments or cryopreservation. The plasmid containing the CAR sequence used in this study was gifted by Wendell Lim (Addgene, 79,125). CAR-L was constructed by loading P2A and pLIRTAC sequentially at the C terminus of CAR. The CAR/CAR-L constructs were packaged into the lentivirus with a triple-plasmid system. The lentivirus was used to infect T cells, and EGFP expression was utilized to identify CAR^+^ T cells. To enhance the transfection efficiency of T cells, His-RetroNectin protein was obtained via the protein purification method, and its concentration was adjusted to 100 μg/ml; this was followed by embedding in a 24-well plate overnight at 4°C in the dark. After addition of the lentivirus, the plate was incubated at 32°C for 4 h, during which the storage solution was removed, and 500 μl of T cell suspension (at a concentration of 1 × 10^6^ cells/ml) was added.

### Flow cytometry

The centrifuged cell precipitate was collected and resuspended at an appropriate concentration. Subsequently, the cells were immediately evaluated for green fluorescence (FITC/EGFP) or red fluorescence (APC/mRFP) levels using flow cytometry. For each experiment, a minimum of 10,000 viable cells per group were included in the analysis, with biological replicates performed at least three times. FlowJo software (V10) was utilized for the analysis of the flow cytometry experimental data. For the identification of purified T cells, the CD3-PE antibody was incubated with the purified T cells at RT for 30 min and then immediately analyzed using flow cytometry to detect the purity of CD3^+^ T cells in the PE channel.

### Integrated density testing, cell imaging, and bioluminescent imaging

Different cells were re-plated into 12-well plates 24 h later (10^5^ cells per well), treated with drugs, and after reaching the expected time point, the integrated fluorescence density of cells in the whole well was detected using a multi-function microplate reader (PerkinElmer, USA). For cell imaging, once the cells reached the desired endpoint, they were incubated with a green lysosomal tracer for 60 min. Afterward, the cells were washed with PBS, stained with Hoechst for 10 min, and then replaced with fresh complete culture medium. Images were captured using a laser confocal microscope (63 ×, ZEISS) in a dark room. For intracranial luminescence imaging in nude mice, D-luciferin potassium salt was intraperitoneally injected into the mice before imaging. Following 10–20 min of isoflurane gas anesthesia, the intensity of intracranial bioluminescence was monitored using a luminescent imaging system (PerkinElmer, USA). The collected bioluminescence data were then analyzed using the processing software of the imaging system.

### Cellular functional experiments

First, the stable cell lines of U251-Vector and U251-OPTN[LIR]A were established. For the CCK-8 assay, cells were cultured in 96-well plates at a density of 10^4^ cells per well, followed by the addition of 10 μl CCK-8 reagent at specific time points. After a 30-min incubation, the OD value at 450 nm was measured using a multiscan spectrum (PerkinElmer, USA). For the cell scratch assay, when the cell density in the 6-well plate reached 80%, cell-free areas were created within the well, and the cell migration status was observed at specific time points. For the Transwell experiment, the chambers of a 24-well plate were coated with matrix gel (Corning, 354,234), 10^4^ cells per well were added, and the cells were cultured for 36 h. After fixing the cells, the bottom of the chamber was photographed to detect the number of migrated cells. In the EdU experiment, cells were cultured to an appropriate density, fixed with paraformaldehyde, stained with EdU (Beyotime, C0078S) for 2 h and with Hoechst 33,342 for nuclear staining, and a fluorescence microscope (Leica) was utilized to detect fluorescent distribution of the cells. For the colony formation assay, adjust the initial cell density to 200 cells per well and culture for 8 days. The fixed cells were then stained with crystal violet for imaging.

### *In vivo* mouse studies

All animal experiments were approved by the Animal Care and Use Committee of Nanchang University (project approval number: NCULAE-20221031007). Healthy nude mice (Gempharmatech, China) were maintained in specific pathogen-free (SPF) conditions and used to establish the intracranial glioblastoma (GBM) models at 4 weeks old. The entire procedure was conducted under isoflurane anesthesia. The injection site was determined (2.5 mm to the right and 1 mm posterior to the anterior fontanel), with each nude mouse receiving an injection of 1 × 10^5^ U251 cells (5 μl). After the injection, the incision was sutured, and the mice were observed for 30 min before being returned to their cages and randomly divided into groups. The growth status of the nude mice was strictly monitored. For the first batch of experimental mice, the injected U251 cells stably expressed luciferase and additionally overexpressed vector, AKTin, or OPTN[LIR]A. For the second batch of mouse experiments, U251-CD19 cells were injected. On the third day after tumor formation, CAR-T/CAR-LT cells were intravenously injected at a dose of 1 × 10^6^ cells per mouse, and the same injection was repeated on day 10 under the same conditions. The mice were euthanized humanely on day 50 when the experiment ended.

Mouse xenograft specimens were fixed, dehydrated, and paraffin-embedded. Tissue sections (3 μm) were deparaffinized, rehydrated, and treated with 3% hydrogen peroxide. Antigen retrieval was performed using EDTA buffer at high temperature for 30 min. Sections were incubated with primary antibody against primary antibody overnight at 4°C, followed by PBS washes and incubation with a secondary antibody for 1 h. Color development was performed using chromogenic substrates. Tissue microarray images were analyzed with ImageScope software (Servicebio, China).

### RNA sequencing

The relevant sequencing information has been uploaded to the Sequence Read Archive (SRA). Accession is PRJNA1168252. T cells stably expressing CAR or CAR-L were activated and subsequently harvested by centrifugation at 300 × g for 5 min. A minimum of 5 × 10^6^ cells was collected per sample. The cell pellets were washed twice with phosphate-buffered saline (PBS), snap-frozen in liquid nitrogen. And then samples were shipped on dry ice to Genewiz Biotechnology for further processing.

### Statistical analysis

All data were analyzed using GraphPad Prism 8 software, and the results were presented as mean ± SEM. For normally distributed data, differences between two groups were compared using a two-tailed Student’s t-test. Survival rates of nude mice were analyzed using the Kaplan-Meier model and two-sided log-rank test. The results of the statistical significance tests are included in the legends of each figure. Whenever possible, the results were confirmed in multiple independent replicate experiments. Male mice matched for age and weight were randomly assigned to the experiments. In our study, apart from CCK-8 and qPCR being technical replicates, the rest of the experimental repeats are biological replicates, with a minimum of three repetitions. A *p*-value < 0.05 was considered to indicate statistically significant differences (*p < 0.05, **p < 0.01, ***p < 0.001, ****p < 0.0001), while ns indicates no significant.

## Supplementary Material

Supplementary_files_R5.docx

## Data Availability

All data necessary to evaluate the conclusions of the paper are in the paper and/or supplementary materials. The RNA-seq data for this project have been uploaded to SRA (https://www.ncbi.nlm.nih.gov/sra/PRJNA1168252). The remaining data are available within the article. No new unique materials or reagents were generated for this study.
