# Supplementary material for: Advancing targeted protein degradation: pLIRTAC’s role in glioma and CAR-T cell therapy
Source: Autophagy. 2026 May 7;22(8):1985–2001. doi: 10.1080/15548627.2026.2668653 (PMC13557589; doi:10.1080/15548627.2026.2668653)
Supplement: Supplementary file 1 [file KAUP_A_2668653_SM0874.docx]

**Table S1.** Sequence information of single gRNA targeting in the designed CRISPR-Cas9 knockout system or shRNA.

| Gene | TargetSeq1 | TargetSeq2 | TargetSeq3 |
| --- | --- | --- | --- |
| ***LC3A*** | **GTCCGCAGCCGACCGCTGTA**AGG | **GTGATCATCGAGCGCTACAA**GGG | **GGTGATCATCGAGCGCTACA**AGG |
| ***LC3B*** | **GAGACTACCTACCGGGATTT**TGG | **CATCCAACCAAAATCCCGGT**AGG | **CTCGCGGCGACACTCACCGA**AGG |
| ***LC3C*** | **AACCAAGTTCCTGGTCCCGC**AGG | **GGTAGTGGAGCGCTACCCCA**GGG | **TGGTTGCGCTCATGCTGACC**AGG |
| ***GABARAP*** | **TTCGAGAAGCGCCGCTCTGA**GGG | **CCGAAAGAAATACCCGGACC**GGG | **TCCGAAAGAAATACCCGGAC**CGG |
| ***GABARAPL1*** | **CTAGTGCCCTCTGACCTTAC**TGG | **GGCAAAGAGCATTACCAGTA**AGG | **TCCGGATTAAGAAGTAGAAC**TGG |
| ***GABARAPL2*** | **ATATTTCGCTCGAATCTTCG**CGG | **TTCGAGCGAAATATCCCGAC**AGG | **TCGAGCGAAATATCCCGACA**GGG |
| ***shTRAF1*** | **CCTTCTACACTGCCAAGTATG** | Undesigned | Undesigned |
| ***ATG5*** | **AACTTGTTTCACGCTATATC**AGG | **TTCCATGAGTTTCCGATTGA**TGG | **TCCGATTGATGGCCCAAAAC**TGG |
| ***RB1CC1*** | **TGAAGATCGGCTCTACGCCC**TGG | **GATTGCTAGCTGTGGCCGAC**TGG | **GATAGACAGTAGACGAATGC**AGG |

**Figure S1.** Validation of degradation efficiency of OPTN[LIR]-EGFP (OPTN[LIR]E) and OPTN[LIR]-mRFP (OPTN[LIR]R). (**A**) Relative ratio of the MFI of different LIRE variants to their corresponding mRFP in 293T cells. (**B**) Integrated density of overexpression of EGFP or different LIRE in 293T and U251 cells. (**C**) GAPDH as an internal reference, the protein expression levels of five LIREs were detected using GFP antibodies. (**D**) The mRNA levels of *EGFP* in multiple U251 cells. (**E**) Schematic diagram of the recombination structure of mOPTN[LIR]R and OPTN[LIR]R. (**F**) Representative micrographs showing the colocalization of LAMP1-EGFP (green, lysosomal marker) and OPTN[LIR]R (red) in the indicated cell lines (63 ×). Scale bar: 20μm. (**G**) Differences in the co-localization correlation between LAMP1-EGFP and mRFP in mOPTN[LIR]R and OPTN[LIR]R groups across various cell lines. (**H**) Integration density of 293T and U251 cells after overexpression of mOPTN[LIR]R or OPTN[LIR]R. (**I**) EGFP and mRFP protein levels in 293T or U251 cells overexpressing WT-mRFP and OPTN^LIR^R using Tubulin as an internal reference. (**J**) Ratio of mRFP to EGFP signals in 293T or U251 cells overexpressing mOPTN[LIR]R and OPTN[LIR]R.

**Figure S2**. OPTN[LIR]E is mainly degraded by the autophagic pathway, and OPTN[LIR]E protein levels can be regulated by Vps34-IN-1, 3MA, and EBSS. (**A and B**) Protein levels and MFI ratio of various recombinant sequences and corresponding mutants in cells. The MFI ratio (**C**) and Integrated density (**D**) of U251 cells with stable OPTN[LIR]E expression were observed after 24 h treatment with DMSO, MG132 (4 μM), CQ (400 nM), 3MA (5 mM), NH_4_Cl (20 mM), and pepA(1 μM). The level of OPTN[LIR]E protein (**E**) and EGFP (**F**) in cells with stable OPTN[LIR]E or EGFP expression after 24 h treatment with DMSO, MG132, CQ, 3MA, NH_4_Cl, and pepA. (**G**) The relative MFI ratio of OPTN[LIR]E to mRFP following treatment with various inhibitors. (**H**) 293T cells stably expressing OPTN[LIR]E were treated with different concentrations of Vps34-IN-1 (left) or 3MA (right), and the changes in OPTN[LIR]E protein levels were detected. The 293T cells with stable OPTN[LIR]E expression were treated with 3MA (**I**) or EBSS (**J**) was detected at different time points. (**K**) Representative micrographs of U251 cells stably expressing OPTN[LIR]R following treatment with various inhibitors (63 ×). Scale bar: 20 μm. (**L**) Comparative analysis of LAMP1-EGFP and mRFP co-localization correlation across different treatment groups in U251-OPTN[LIR]R cells.

**Figure S3**. GABARAPL2 and LC3B can significantly regulate the degradation level of OPTN[LIR]E, and degradation of OPTN[LIR]E requires autophagy pathway components, including RB1CC1 and ATG8 family proteins. (**A**) Verification of knockout efficiency of ATG8 (autophagy related 8)-family proteins in 293T and U251 cells. Red indicates the gRNAs with the highest knockout efficiency. The Ctrl group represents cells that have not been treated, and the sgVector group represents cells that stably express the empty vector corresponding to the experimental group. OPTN[LIR]E protein levels (**B**) and quantitative data analysis (**C**) in cells after ATG8-family proteins were knocked out. (**D**) Verification of knockout efficiency of *RB1CC1* and *ATG5* in U251 and 293T cells. Red indicates the gRNAs with the highest knockout efficiency. (**E**) Co-immunoprecipitation to detect protein interactions. (**F**) Integration density in cells after knocking out the corresponding protein.

**Figure S4**. OPTN[LIR]G can significantly degrade intracellular EGFP through the same degradation pathway as OPTN[LIR]E. (**A**) Effect of GFPnanobody, mOPTN[LIR]G, and OPTN[LIR]G on EGFP protein degradation in cells. (**B**) Green fluorescence signal intensity detected after GFPnanobody, mOPTN[LIR]G, or OPTN[LIR]G expression in 293T-EGFP cells. (**C**) Changes in EGFP protein levels after treatment of U251/293T-EGFP cells with the above drugs or EBSS combined with OPTN[LIR]G. (**D**) 293T-EGFP cells were treated with OPTN[LIR]G combined with multiple drugs to detect MFI. (**E**) Changes in the degradation efficiency of OPTN[LIR]G to EGFP after knocking out certain ATG8 family proteins. (**F**) Cell images of U251/293T-YFP cells treated with mOPTN[LIR]G or OPTN[LIR]G for 24 h (63×). Scale Bar: 20 μm. (**G**) Comparative analysis of LAMP1-EGFP and YFP co-localization correlation. The MFI (**H**) and EGFP protein levels (**I**) of 293T-EGFP cells were delivered with different concentrations of His-TAT-OPTN[LIR]G. His-TAT-mOPTN[LIR]G/OPTN[LIR]G detection is based on His-tag flow cytometry antibody detection of intracellular proteins. (**J**) His-TAT-OPTN[LIR]G was directly supplemented into U251-EGFP cell cultures, followed by an 8-hour co-incubation with different inhibitors. (**K**) Effect of ATG8 family protein knock-out on His TAT-OPTN[LIR]G knock-down efficiency of EGFP. (**L**) The green MFI of U251-EGFP cells with *GABARAPL2* or *LC3C* protein knocked out and His-TAT-OPTN[LIR]G added.

**Figure S5**. OPTN[LIR]A inhibits the occurrence and development of glioma cells after targeted degradation of endogenous AKT1 and p-AKT, and EGFP, AKT1, and p-AKT can be significantly degraded by exogenous pLIRTAC. (**A**) Changes in protein levels caused by the application of different pLIRTAC molecules to U251 cells. (**B**) Left, overall docking poses of AKT1 with AKTin, and KDM5B with KDM5B-PBD. Right, magnified views of the corresponding interaction interfaces. Hydrogen bonds are depicted as yellow dashed lines, with bond lengths (in Å) and the participating amino acid residues labeled. (**C**) The difference in proliferation ability between 251-OPTN[LIR]A, 251-AKTin, and control cells. (D) Migration status of 251-OPTN[LIR]A, 251-AKTin, and control cells. Scale bar: 500 μm. (**E**) Invasion level of 251-OPTN[LIR]A, 251-AKTin, and control cells. Scale bar: 100 μm. (**F**) The ratio of active proliferating cells to total cells. Scale bar: 100 μm. (**G**) Differences in the number of monoclonal colonies between 251-OPTN[LIR]A and 251-Vector cells. (**H**) AKT protein levels in U251 cells at different time points after the addition of His-TAT-OPTN[LIR]A or His-TAT-mOPTN[LIR]A. (**I**) AKT and p-AKT protein levels in U251 cells at different time points after the addition of His-TAT-OPTN[LIR]A or His-TAT-AKTin. (**J**) Protein levels of AKT1 and p-AKT in different cell groups after 3MA treatment. (**K**) Weight changes in each group of mice. (**L**) Immunohistochemical analysis of glioma xenografts in the mouse brain. Left, representative IHC images (60×) of the indicated markers in intracranial glioma tissues from nude mice. Scale bar: 100 μm. Right, quantitative analysis of the H-Score assessed in three randomly selected regions per tumor.

**Figure S6**. pLIRTAC targets AKT1 in glioma cells *in vivo*, and the pLIRTAC can enhance the persistence of CAR-T cells. (**A**) Experimental timeline of glioma mice treated by intrathecal injection. (**B**) Weight changes in mice treated with CAR-T/CAR-LT cells for glioma *in vivo*. (**C**) The proportion of CD3^+^ T cells in PBMC before (Top) and after (Bottom) purification. (**D**) Top, RNF31 protein levels in different cells. HSDA: RNF31 single-domain antibody. Bottom, The effects of OPTN[LIR]G, OPTN[LIR]A, and OPTN[LIR]H on endogenous proteins in T cells. (**E**) Top, RNF31 protein levels in CAR-T cells and CAR-LT cells. Bottom, RNF31 protein levels in CAR-LT cells after 3MA treatment. (**F**) KEGG analysis shows a rich factor in pathway enrichment. (**G**) Western blot displayed the protein levels of RNF31 and TRAF1 in CAR-LT cells. (**H**) Effect of overexpression of TRAF1 on apoptosis of CAR-T cells. (**I**) Protein level changes of RNF31 and TRAF1 in CAR-LT cells after 3MA treatment. (**J**) The sustained cytolytic activity of CAR-LT cells against target cells is significantly impaired after 3MA treatment.
